# Exponential Decay of Truncated Correlations for the Ising Model in any Dimension for all but the Critical Temperature

**DOI:** 10.1007/s00220-019-03633-y

**Published:** 2019-11-28

**Authors:** Hugo Duminil-Copin, Subhajit Goswami, Aran Raoufi

**Affiliations:** 1 Université de Genève, Geneva, Switzerland; 2 Institut des Hautes Études Scientifiques, Bures-sur-Yvette, France; 3 ETH Zurich, Zurich, Switzerland

## Abstract

The truncated two-point function of the ferromagnetic Ising model on ${\mathbb {Z}}^d$ ($d\ge 3$) in its pure phases is proven to decay exponentially fast throughout the ordered regime ($\beta >\beta _c$ and $h=0$). Together with the previously known results, this implies that the exponential clustering property holds throughout the model’s phase diagram except for the critical point: $(\beta ,h) = (\beta _c,0)$.

## Introduction

### Exponential decay of truncated correlations of the Ising model

In addition to its original presentation as a model for the phase transition in ferromagnets, the Ising model has attracted attention from a variety of perspectives. These range from studies of phase transitions exhibited by the equilibrium states to the study of cutoff phenomena and transitions in stochastic processes given for instance by Glauber dynamics and Metropolis algorithms [LS13]. Also, universality of critical phenomena in the Ising model justifies the fact that the theory of the Ising model provides information also about many other systems.

As is well known, sufficiently far from phase transitions, systems of statistical physics exhibit exponential relaxation of truncated correlations [DS87], in both the equilibrium and the dynamical sense. It is more challenging to narrow the range of exceptions to a set of points, or lines, in the model’s phase space. The main result in this article completes that task for the *d*-dimensional nearest-neighbor ferromagnetic Ising model. The results extend to finite-range Ising models, but we choose to focus on the nearest-neighbor case for simplicity.

To set the notation, let us recall the definition of the model on a graph *G* with vertex-set *V* and edge-set *E*. Associated with the graph’s vertex-set is a collection of binary variables $\sigma =(\sigma _x:x\in V)$, with $\sigma _x\in \{-1,1\}$. The system’s Hamiltonian is given by the function

1.1 $$\begin{aligned} H_ {G,h}(\sigma )~:=~ -\sum _{x\in V} h \sigma _x \ -\ \sum _{\{x,y\}\subset V } J_{x,y} \sigma _x\sigma _y\,, \end{aligned}$$

with the magnetic field *h* and coupling constants $J_{x,y}$. In the case on which we focus here, *G* equals $\mathbb {Z}^d$ (the graph is the regular *d*-dimensional lattice) and

$$\begin{aligned} J_{x,y} \ = \ {\left\{ \begin{array}{ll} 1 &{}\quad \text { if }\{x,y\}\in E, \\ 0 &{}\quad \text { otherwise, } \end{array}\right. } \end{aligned}$$

which corresponds to nearest-neighbor ferromagnetic interactions.

On finite graphs, the Gibbs equilibrium states at inverse temperature $\beta \in (0, \infty )$ are given by probability measures on the space of configurations under which the expected value of a function $f:\{-1,1\}^{V}\rightarrow {\mathbb {R}}$ is

$$\begin{aligned} \langle f\rangle _{ G,\beta ,h}=\frac{1}{Z( G,\beta ,h)}\sum _{\sigma \in \{-1,1\}^{V}}f(\sigma )\exp [-\beta H_ {G,h}(\sigma )]\, , \end{aligned}$$

where the sum is normalized by the partition function $Z( G,\beta ,h)$ so that $\langle 1\rangle _{ G,\beta ,h}=1$. Examples of Gibbs measures on $\mathbb {Z}^d$ can be constructed as weak limits of the finite volume Gibbs measures on finite subgraphs $G\subset \mathbb {Z}^d$ which locally converge to $\mathbb {Z}^d$. We denote the measure thus obtained by $\langle \cdot \rangle _{\beta ,h}$. Also, one defines

1.2 $$\begin{aligned} \langle \cdot \rangle _{\beta }^+ \ = \ \lim _{h\searrow 0} \langle \cdot \rangle _{\beta ,h} \end{aligned}$$

where the limit is meant in the “weak sense” (i.e. for the expectation values of local functions of the spins). Convergence can be deduced by monotonicity arguments based on correlation inequalities, by which one may also establish the existence of $\beta _c=\beta _c(\mathbb {Z}^d)\in [0,\infty ]$ such that

1.3 $$\begin{aligned} 0 \le \beta < \beta _c\Rightarrow & {} \langle \sigma _x\rangle _{\beta }^+ \ = \ 0,\quad \forall x\in \mathbb {Z}^d, \nonumber \\ \beta> \beta _c\Rightarrow & {} \langle \sigma _x\rangle _{\beta }^+ \ >\ 0,\quad \forall x\in \mathbb {Z}^d. \end{aligned}$$

For a given Gibbs measure $\langle \cdot \rangle$, in finite or infinite volume, the truncated two-point correlation function is defined as:

$$\begin{aligned} \langle \sigma _0;\sigma _x\rangle :=\langle \sigma _0\sigma _x\rangle -\langle \sigma _0\rangle \langle \sigma _x\rangle . \end{aligned}$$

For $\beta > \beta _c$, there exists a spin-flip symmetric equilibrium state with long-range order, for which the truncated correlations do not decay to zero. However, the relevant question is the rate of decay of the pure state $\langle \cdot \rangle _{\beta }^+$ and its symmetric image $\langle \cdot \rangle _{\beta }^-$. The main result of this article is the following.

#### Theorem 1.1

For the nearest-neighbor Ising model on $\mathbb {Z}^d$ in dimension $d \ge 3$, for any $\beta >\beta _c$ there exists $c = c(\beta ,d) > 0$ such that for every $x,y\in \mathbb {Z}^d$,

1.4 $$\begin{aligned} 0\le \langle \sigma _x;\sigma _y\rangle _{\beta }^+\le \exp [- c\Vert x-y\Vert ]. \end{aligned}$$

The previous result holds for any extremal translation invariant Gibbs state, since by [Bod05, Rao17], those are given by $\langle \cdot \rangle _\beta ^+$ and $\langle \cdot \rangle _\beta ^-$. As mentioned above, the theorem would extend to finite-range interactions, but we prefer to focus on this context for simplicity of notations. Jointly with the previously known results, this completes the proof that for the nearest-neighbor Ising model in any dimension it is only at the critical point $(h,\beta ) = (0,\beta _c)$ that the pure state’s truncated two-point function fails to decay exponentially fast. The aforementioned statement which Theorem 1.1 supplements include:

1. At any $h\ne 0$ the limiting state is analytic in *h* and $\beta$, and it exhibits exponential decay of suitably truncated correlations. This was proven by Lebowitz and Penrose in [LP68] using an argument which drew on the model’s Lee-Yang property [LY52], or using the random-current representation.
2. For $h=0$ and $\beta < \beta _c$ the exponential decay in arbitrary dimension was established in [ABF87] (see also [DT16] for an alternative proof).
3. In the converse direction: the vanishing of the spontaneous magnetization at $(h,\beta ) = (0,\beta _c)$ for the nearest neighbor model in any dimension [ADCS15] together with the lower bound
  1.5 $$\begin{aligned} \sum _{\Vert x\Vert _\infty =R}\langle \sigma _0\sigma _x\rangle _{\beta _c}\ \ge \ 1 \, , \end{aligned}$$
  which was established by Simon [Sim80], imply that for any $d\ge 2$ at the critical point the truncated two-point function does not decay exponentially fast.
4. And, to mention a last result: the special case of $d=2$ is analyzable through Onsager’s exact solution [MW73] or using the Kramers-Wannier duality (the decay of truncated correlations can be obtained via the decay of correlations in the high-temperature dual Ising model).

Let us add that the truncated two-point function offers a bound on the decay of more general correlations which follows easily from the following lemma (whose proof follows from the switching lemma; see the discussion in Sect. 2.2). For a set of vertices *A*, set $\sigma _A:=\prod _{x\in A}\sigma _x$.

#### Lemma 1.2

For every finite graph *G*, every $\beta >\beta _c$ and every two disjoint sets of vertices *A* and *B*, we have that

1.6 $$\begin{aligned} 0 \le \langle \sigma _A\sigma _B \rangle ^+_{G,\beta } - \langle \sigma _A \rangle ^+_{G,\beta } \langle \sigma _B\rangle ^+_{G,\beta } \le \, 2^{| A| + | B| - 4} \, \sum _{\begin{array}{c} a\in A\\ b\in B \end{array}} \langle \sigma _a;\sigma _b \rangle ^{+}_{G,\beta } \, . \end{aligned}$$

### Mixing of the Fortuin–Kasteleyn representation of the Ising model

Theorem 1.1 will be derived by studying a related model, called the *Fortuin–Kasteleyn (FK) percolation*, or random-cluster model. FK percolation is one of the most classical generalizations of Bernoulli percolation and electrical networks. This model was introduced by Fortuin and Kasteleyn in [FK72] and since then has been the object of intense studies, both physically and mathematically.

A *percolation configuration* on a graph $G=(V,E)$ is an element $\omega =(\omega _e:e\in E)$ in $\{0,1\}^{E}$. An edge *e* is said to be *open* (in $\omega$) if $\omega _e=1$, otherwise it is *closed*. A configuration $\omega$ can be seen as a subgraph of *G* with vertex-set *V* and edge-set $\{e\in E:\omega _e=1\}$. A *cluster* is a connected component of the graph $\omega$. Below, we will write $x\leftrightarrow y$ if *x* and *y* are in the same cluster, and $x\leftrightarrow \infty$ if *x* is in an infinite cluster.

Fix $p\in [0,1]$ and $q\ge 1$. Let *G* be a finite subgraph of $\mathbb {Z}^d$ and $\xi$ a configuration on $\mathbb {Z}^d$. Let $\phi _{G,p,q}^\xi$ be the measure on percolation configurations $\omega$ on *G* defined by

$$\begin{aligned} \phi _{G,p,q}^\xi (\omega )=\frac{1}{Z^\xi (G,p,q)}\big (\tfrac{p}{1-p}\big )^{|\omega |} q^{k_\xi (\omega )}, \end{aligned}$$

where $|\omega |:=\sum _{e\in E}\omega _e$ and $k_\xi (\omega )$ is the number of clusters intersecting *G* of the percolation configuration ${\overline{\omega }}$ on $\mathbb {Z}^d$ defined by $\overline{\omega }_e=\omega _e$ if $e\in E$, and $\xi _e$ if $e\notin E$, and $Z^\xi (G,p,q)$ is a normalizing constant making the total mass of the measure equal to 1. We refer to $\xi$ as the *boundary condition* of $\phi _{G,p,q}^\xi$. In the particular case when $\xi \equiv 1$ (or 0) we denote the corresponding measure by $\phi _{G,p,q}^1$ (respectively $\phi _{G,p,q}^0$) and call the corresponding boundary condition as *wired* (respectively *free*).

The FK-percolation model with cluster-weight $q=2$ is related to the Ising model via the Edwards–Sokal coupling (see next section) and is therefore referred to in this article as the *FK-Ising model*. When $q=2$, it was proved in [Bod06, Rao17] that for every $p\in [0,1]$, there exists a unique infinite-volume measure $\phi _{p,2}$ which is the weak limit of measures $\phi _{G,p,2}^\xi$ as *G* exhausts $\mathbb {Z}^d$. Furthermore, there exists a constant $p_c=p_c(d)$ such that $\phi _{p,2}[0\leftrightarrow \infty ]$ is equal to 0 for every $p<p_c$, and is strictly positive for every $p>p_c$. We refer to [Gri06] for a justification that this limit exists.

Below and in the rest of this paper, we focus on the case $q=2$ and drop it from the notation. We denote $x + [-n, n]^d \cap \mathbb {Z}^d$ by $\Lambda _n(x)$ and the set of edges between two vertices of $\Lambda _n(x)$ by $E_n(x)$. In the particular case when $x = 0$, we write $\Lambda _n$ and $E_n$ respectively. The *boundary* of $\Lambda _n(x)$, denoted as $\partial \Lambda _n(x)$, is defined as the set of all vertices in $\Lambda _n(x)$ which have a neighbor in $\mathbb {Z}^d {\setminus } \Lambda _n(x)$. Theorem 1.1 is a consequence of the following exponential mixing property.

#### Theorem 1.3

(Exponential mixing). For every $d\ge 3$ and $p>p_c$, there exists a constant $c>0$ such that for every $n\ge 1$,

1.7 $$\begin{aligned} |\phi _{p}[A\cap B] - \phi _{p}[A]\phi _{p}[B]| \le \exp (-cn), \end{aligned}$$

where *A* and *B* are any two events depending on edges in $E_{n}$ and outside $E_{2n}$ respectively.

Before discussing the proof of this theorem, let us explain how it implies Theorem 1.1.

#### Proof of Theorem 1.1

Fix $\beta >\beta _c$ and set $p:=1-e^{-2\beta }>p_c$. The Edwards–Sokal coupling [see (2.6) in the next section] gives that for every $x\in \mathbb {Z}^d$,

1.8 $$\begin{aligned} \langle \sigma _0; \sigma _x \rangle _{\beta }^+&=\langle \sigma _0\sigma _x\rangle _{\beta }^+-\langle \sigma _0\rangle _\beta ^+\langle \sigma _x\rangle _\beta ^+= \phi _{p}[0\leftrightarrow x] - \phi _{p}[0\leftrightarrow \infty ]\phi _{p}[x\leftrightarrow \infty ]. \end{aligned}$$

Assuming that *x* is at a graph distance of at least 4*n* of the origin, this implies that

1.9 $$\begin{aligned} \langle \sigma _0; \sigma _x\rangle _{\beta }^+&\le \phi _{p}[0\leftrightarrow \partial \Lambda _n,x\leftrightarrow \partial \Lambda _n(x)] - \phi _{p}[0\leftrightarrow \partial \Lambda _n]\phi _{p}[x\leftrightarrow \partial \Lambda _n(x)]+2e^{-c'n}\nonumber \\&\le \mathrm {e}^{-cn}+2\mathrm {e}^{-c'n}, \end{aligned}$$

where in the second line, we used Theorem 1.3, and in the first, the fact that for $p>p_c$, there exists $c'>0$ such that for every $n\ge 1$,

1.10 $$\begin{aligned} \phi _{p}[0\leftrightarrow \partial \Lambda _n,0\nleftrightarrow \infty ]\le \mathrm {e}^{-c'n}. \end{aligned}$$

(This fact follows from Theorem (5.104) of [Gri06] when combined with the result of [Bod05].) $\quad \square$

Let us remark that the exponential decay (1.10) for the size of finite clusters in the supercritical FK-Ising model does not directly imply the exponential decay of truncated two-point functions for the Ising model, since the first term on the right of (1.9) involves correlations between the events that $0\leftrightarrow \partial \Lambda _n$ and $x\leftrightarrow \partial \Lambda _n(x)$, and that these correlations could *a priori* be large.

Let us also remark that our method actually gives a better bound on the error term in Theorem 1.3 than $\exp (-cn)$. Namely, we obtain that

1.11 $$\begin{aligned} |\phi _p[A\cap B]-\phi _p[A]\phi _p[B]|\le \exp (-cn)\max _\xi \phi _{\Lambda _n, p}^\xi [A]\phi _p[B]\,. \end{aligned}$$

This is stronger than the *weak mixing* property for FK percolation measures which is obtained by replacing $\max _\xi \phi _{\Lambda _n, p}^\xi [A]$ with 1 but weaker than the *ratio weak mixing* property where we want to get rid of the maximum over boundary conditions. However our proof of Theorem 1.3 (see, e.g., (3.4)) also implies that $\phi _p$ has the so-called *exponentially bounded controlling regions* in the sense of [Ale98, p. 455]. Then the ratio weak mixing property of $\phi _p$ follows from (1.11) and Theorem 3.3. in [Ale98]. For potential application in future works we present it here as a corollary of Theorem 1.3.

#### Corollary 1.4

(Ratio weak mixing). For every $d\ge 3$ and $p>p_c$, there exists a constant $c>0$ such that for every $n\ge 1$,

1.12 $$\begin{aligned} |\phi _{p}[A\cap B] - \phi _{p}[A]\phi _{p}[B]| \le \exp (-cn)\phi _{p}[A]\phi _{p}[B], \end{aligned}$$

where *A* and *B* are any two events depending on edges in $E_{n}$ and outside $E_{2n}$ respectively.

### Idea of the proof

The core of the proof will be the derivation of the following proposition.

#### Proposition 1.5

There exists $c>0$ such that for every integer *N* that is divisible by 4,

$$\begin{aligned} \max _{e \in E_{N/4}} \phi _{\Lambda _{N}, p}^1[\omega _e] - \phi _{\Lambda _{N}, p}^0[\omega _e] \le \exp [-c(\log N)^{1 + c}]\,. \end{aligned}$$

The important feature of the upper bound above is that it beats any inverse polynomial. This proposition, together with a coarse-grained argument inspired by Pisztora, implies the exponential mixing. While the Ising model can be approached through a number of graphical representations (low and high temperature expansions, FK-Ising, random current etc.), which have been used separately in a variety of results, the argument presented here relies in a crucial way on the combination of two such representations: the random current and the FK-Ising. The random current representation is used to rewrite the difference between $\phi _{\Lambda _{N}, p}^1[\omega _e]$ and $\phi _{\Lambda _{N}, p}^0[\omega _e]$ in terms of the probabilities of non-intersection for currents in a duplicated system of currents. Then, FK-Ising is used to show that this duplicated system of currents is very well-connected, and that the probability of long paths of currents not being connected is quite small.

At different stages of the proof (already in the proof of Theorem 1.1 above), essential use is made of the very helpful result of Bodineau [Bod05] stating that for any $d\ge 3$ the critical parameter $p_c$ coincides with the so-called *slab percolation*. This result is combined with the result [Pis96] to implement a coarse-grain argument inspired by Pisztora renormalization. This is used to prove two facts: boxes are connected with excellent probability in the supercritical FK-Ising model, and Theorem 1.3 follows from Proposition 1.5.

### Open problems

Corollary 1.4 falls short of the *ratio-strong mixing property* related to the phenomenon of *boundary phase transition* for Ising models (see [MOS94]). Although this stronger property is absent for Ising models in dimensions larger than 2 at low temperature, it is expected to hold in the entire subcritical phase. More precisely, one would like to prove:

$$\begin{aligned} |\phi _p[A\cap B] - \phi _{p}[A]\phi _{p}[B]|\le \exp (-c \, d_{A, B})\phi _{p}[A]\phi _{p}[B] \end{aligned}$$

where $d_{A, B}$ is the distance between the supports of the events *A* and *B*.

Another important improvement would be to understand the case of the Potts models with $q\ge 3$ colors. While the $\beta <\beta _c$ was recently treated in every dimension in [DRT17], the study of the $\beta >\beta _c$ regime is still very limited. In [DT17], a partial result going in the direction of the equivalent of [Bod05] for Potts model was obtained. We refer to the paper for details on open questions and conjectures. Bodineau’s result being the key to our argument (not to mention the heavy use of the random-current representation, which itself is not available for the Potts model), we believe that the exponential decay of correlations would be even harder to obtain than the open problems mentioned in [DT17].

*Organization* The paper is organized as follows. In the next section, we recall some background. In Sect. 3, we present the coarse-graining arguments relying on Pisztora’s technique. In Sect. 4, we prove Proposition 1.5, conditionally on two technical statements which are proved in Sect. 5.

## Background

### The FK-Ising model

We will use a few properties of the FK-Ising model that we recall now. For details and proofs, we direct the reader to [Gri06, Dum17].

*Spatial Markov property*

Let $H\subset G$ be two finite subgraphs of $\mathbb {Z}^d$ with respective edge-sets *E* and *F*. A configuration $\omega$ on *G* may be viewed as a configuration on *H* by taking its restriction $\omega _{|E}$. The restriction of the configuration $\omega$ to edges of $F{\setminus } E$ induces boundary conditions on *G*. Namely, the spatial Markov property states that for any *p*, *q* and any configuration $\xi$,

2.1 $$\begin{aligned} \phi _{G,p}^\xi (\omega _{|E}=\cdot \,|\omega _e=\xi _e,\forall e\in F{\setminus } E)=\phi _{H,p}^{\xi }(\cdot ). \end{aligned}$$

The spatial Markov property implies the following finite-energy property: for every $\xi$,

2.2 $$\begin{aligned} \tfrac{p}{2-p}\le \phi _{\{e\},p}^\xi [\omega _e]\le p. \end{aligned}$$

*Stochastic ordering for *$q\ge 1$. For any finite graph *G*, the set $\{0,1\}^{E}$ has a natural partial order. An event *A* is *increasing* if for every $\omega \le \omega '$, $\omega \in A$ implies $\omega '\in A$. The FK-Ising model satisfies the following properties. Fix $p\in [0,1]$ and $\xi \le \xi '$,

1. (FKG inequality) For every two increasing events *A* and *B*,
  2.3 $$\begin{aligned} \phi _{G,p}^\xi [A\cap B]\ge \phi _{G,p}^\xi [A]\phi _{G,p}^\xi [B]. \end{aligned}$$
2. (Comparison between boundary conditions) For every increasing event *A*,
  2.4 $$\begin{aligned} \phi _{G,p}^{\xi '}[A]\ge \phi _{G,p}^\xi [A]. \end{aligned}$$

This last condition, together with (2.1), enables one to construct measures $\phi ^1_{p}$ and $\phi ^0_{p}$ in $\mathbb {Z}^d$ as weak limits of measures with free and wired boundary conditions in finite volume. It was proved in [Bod06] (see also [Rao17]) that $\phi ^1_p=\phi ^0_p$ for every $p\ne p_c$ (see [ADCS15] for the case $p=p_c$), and this is the reason why we refer to the infinite-volume measure as simply $\phi _p$.

#### Remark 2.1

We will often consider couplings between two FK-Ising measures. In this case, we will use the spatial Markov property and the FKG inequality applied to *both configurations at the same time*, two properties that we will call *joint Markov property* and *joint FKG inequality*. These properties will be justified by the standard spatial Markov property and the FKG inequality combined with the special constructions of these measures. Since the justification is classical, we will omit it in this article.

*Edwards–Sokal coupling*

We will use the Edwards–Sokal coupling both in finite and infinite volume. On a finite graph, the coupling goes as follows. Consider $\beta$ and *p* related by $p=1-e^{-2\beta }$. Consider a configuration $\omega$ sampled according to $\phi ^0_{G,p}$ and assign to each cluster ${\mathcal {C}}$ of $\omega$ a spin $\sigma _{{\mathcal {C}}}$ in $\{-1,+1\}$ uniformly and independently for each cluster. Then, set $\sigma _x=\sigma _{{\mathcal {C}}}$ for every $x\in {\mathcal {C}}$. As a direct consequence of this coupling, one obtains that

2.5 $$\begin{aligned} \langle \sigma _A\rangle _{G,\beta }=\phi _{G,p}^0[{\mathcal {F}}_A], \end{aligned}$$

where ${\mathcal {F}}_A$ is the event that every cluster of $\omega$ intersects an even number of times the set *A*. Note that when $A=\{x,y\}$, this translates into $\langle \sigma _x\sigma _y\rangle _{G,\beta }=\phi _{G,p}^0[x\leftrightarrow y]$.

We will also use the coupling in infinite volume. In this case, one can consider $\phi _p$ and assign a spin to each one of the finite clusters at random as explained previously, and a spin $+$ to the infinite clusters (there is in fact at most one such cluster). One then obtains the measure $\langle \cdot \rangle _{\beta }^+$. Altogether, we deduce from this representation that

2.6 $$\begin{aligned} \langle \sigma _x\rangle _{\beta }^+=\phi _{p}[x\leftrightarrow \infty ]. \end{aligned}$$

We have in particular that $\beta _c=\tfrac{1}{2}\log (1-p_c)$.

*Griffiths inequality* The monotonicity properties of the FK-Ising model imply the following two classical inequalities, which will be very useful: for every sets of vertices *A* and *B*,

2.7 $$\begin{aligned} \langle \sigma _A\sigma _B\rangle _{G,\beta }^+\ge \langle \sigma _A\rangle _{G,\beta }^+\langle \sigma _B\rangle _{G,\beta }^+ \end{aligned}$$

and, if exceptionally we consider the model with arbitrary coupling constants *J* (we refer to *J* in the notation by writing $\langle \cdot \rangle _{G,J,\beta }$ for the measure), we have that for every coupling constants $J\ge J'\ge 0$,

2.8 $$\begin{aligned} \langle \sigma _A\rangle _{G,J,\beta }\ge \langle \sigma _A\rangle _{G,J',\beta }. \end{aligned}$$

### The random-current representation

We will also use the random-current representation in several places. A *current* configuration $\mathbf {n}$ on a graph *G* with vertex-set *V* and edge-set *E* is an integer valued function on *E*, i.e. a function $\mathbf {n}: E \mapsto {\mathbb {Z}}_+$. A *source* of $\mathbf {n}=(\mathbf {n}(x,y):\{x,y\}\in E)$ is a vertex *x* for which $\Delta _x(\mathbf {n}):=\sum _{y\in V:y\sim x}{\mathbf {n}}(x,y)$ is odd. The set of sources of $\mathbf {n}$ is denoted by $\partial \mathbf {n}$. The random current configuration’s *weight*, at specified $\beta >0$, is given by

2.9 $$\begin{aligned} w_{\beta }(\mathbf {n}):=\prod _{\{x,y\}\in E}\frac{\beta ^{\,{\mathbf {n}}(x,y)}}{{\mathbf {n}}(x,y)!}. \end{aligned}$$

For every finite subgraph *G* of $\mathbb {Z}^d$, we also construct a graph $G^+=(V^+,E^+)$ with $V^+=V\cup \{{\mathfrak {g}}\}$, where ${\mathfrak {g}}$ is called the *ghost* vertex, and $E^+$ is the union of *E* together with as many edges $\{x,{\mathfrak {g}}\}$ as edges between *x* and a vertex of $\mathbb {Z}^d$ outside of *G*. Note that there can be multiple edges between two given vertices in $G^+$, but that only vertices on the boundary (i.e. the vertices neighboring a vertex in $\mathbb {Z}^d {\setminus } V$) of *G* can be connected to the ghost vertex.

Correlations of the Ising model can be expressed in terms of the random-current representation via the following formula: for every $A\subset V$,

2.10 $$\begin{aligned} \langle \sigma _A\rangle _{G,\beta }:=\frac{\displaystyle \sum _{\mathbf {n}\in \mathbb {Z}_+^E:\partial \mathbf {n}=A}w_\beta (\mathbf {n})}{\displaystyle \sum _{\mathbf {n}\in \mathbb {Z}_+^E:\partial \mathbf {n}=\emptyset }w_\beta (\mathbf {n})}\qquad \text {and}\qquad \langle \sigma _A\rangle _{G,\beta }^+:=\frac{\displaystyle \sum _{\mathbf {n}\in \mathbb {Z}_+^{E^+}:\partial \mathbf {n}\cap V=A}w_\beta (\mathbf {n})}{\displaystyle \sum _{\mathbf {n}\in \mathbb {Z}_+^{E^+}:\partial \mathbf {n}\cap V=\emptyset }w_\beta (\mathbf {n})}. \end{aligned}$$

Note that the random-current representation of spin correlations for free boundary conditions involve currents on *G*, while those for plus boundary conditions involve currents on $G^+$.

The great utility of the random current representation results from a switching symmetry, whose roots lie in a combinatorial identity of [GHS70]. Using this symmetry, the Ising phase transition was presented in [Aiz82] as a phenomenon of percolation in a system of current loops. Resulting relations have been instrumental in shedding light on the critical behavior of the model in various dimensions [Aiz82, ABF87, AF86, ADCS15, ADTW18]. We do not wish to state the switching lemma here and refer to the corresponding literature. Rather, we present the applications we will need for our study.

To express various correlation functions (of finite systems) in terms of probabilities for systems of currents with prescribed sources, we introduce the probability measure ${\mathbb {P}}^{A}_{G}$ on currents $\mathbf {n}\in {\mathbb {Z}}_+^E$ with $\partial \mathbf {n}=A$ by the formula

2.11 $$\begin{aligned} \mathbb P^{A}_{G}[\{\mathbf {n}\}]:=\frac{w_{\beta }(\mathbf {n})}{\displaystyle \sum _{\mathbf{m}\in \mathbb {Z}_+^{E}:\partial \mathbf{m}=A}w_{\beta }(\mathbf{m})}\,. \end{aligned}$$

Similarly, one defines ${\mathbb {P}}^A_{G^+}$ on currents $\mathbf {n}\in {\mathbb {Z}}_+^{E_+}$ with $\partial \mathbf {n}\cap V=A$. Let $\mathbb P^{A,\emptyset }_{G^+,G}$ (resp. ${\mathbb {P}}^{A,\emptyset }_{G^+,G^+}$) denote the law of two independent currents with respective laws ${\mathbb {P}}^A_{G^+}$ and ${\mathbb {P}}^\emptyset _{G}$ (resp. $\mathbb P^\emptyset _{G^+}$). The key relation of interest, which, at the risk of repeating ourselves, is a consequence of the switching lemma, is the following:2.12where the event on the left denotes the fact that *x* and *y* are connected by a path $x=x_0\sim \dots \sim x_m=y$ of neighboring vertices of *G* such that $(\mathbf {n}_1+\mathbf {n}_2)(x_i,x_{i+1})>0$ for every $0\le i<m$. Sometimes, we will consider two sets *X* and *Y* instead of *x* and *y*. By this, we mean that some vertex in *X* is connected to some vertex in *Y*.

A special case of this relation consists in choosing $A=\{x,y\}$, which gives2.13We conclude the section by proving Lemma 1.2.

#### Proof of Lemma 1.2

Let *G* be a finite subgraph of $\mathbb {Z}^d$. For $S \subset V^+$, let ${\mathcal {C}}_{\mathbf n}(S)$ denote the set of all vertices in $G^+$ which are connected to *S* by $\mathbf n$. Since *A* and *B* are disjoint, the switching lemma implies that

$$\begin{aligned} \langle \sigma _A\sigma _B \rangle ^{+}_{G, \beta } - \langle \sigma _A \rangle ^{+}_{G, \beta } \langle \sigma _B\rangle ^{+}_{G, \beta } = \langle \sigma _{A \cup B} \rangle ^{+}_{G, \beta } \, {\mathbb {P}}^{A \cup B,\emptyset }_{G^+,G^+}[ 1 - {\mathbb {I}}_{{\mathcal {F}}_B}], \end{aligned}$$

where ${\mathcal {F}}_B$, when |*B*| is even, is the event that every connected component of $\mathbf n_1 + \mathbf n_2$ contains an even number of vertices from *B*. When |*B*| is odd, ${\mathcal {F}}_B$ is the event that every connected component of $\mathbf n_1 + \mathbf n_2$ contains an even number of vertices from $B \cup \{ \mathfrak {g}\}$. Hence, to prove the lemma it suffices to demonstrate the inequality

2.14 $$\begin{aligned} \sum _{\begin{array}{c} \partial \mathbf n_1 \cap V = A \cup B\\ \partial \mathbf n_2 = \emptyset \end{array}} \,&\omega _\beta (\mathbf n_1) \, \omega _\beta (\mathbf n_2) \, {\mathbb {I}} [\mathbf n_1 + \mathbf n_2 \notin {\mathcal {F}}_B] \nonumber \\&\le \sum _{\begin{array}{c} A' \subset A \\ | A'| \text { is odd } \end{array}} \sum _{\begin{array}{c} B' \subset B \\ | B'| \text { is odd } \end{array}} \sum _{\begin{array}{c} a \in A'\\ \, b \in B' \end{array}} \sum _{\begin{array}{c} \partial \mathbf n_1 = \{a ,b \}\\ \partial \mathbf n_2 = \emptyset \end{array}} \, \omega _\beta (\mathbf n_1) \, \omega _\beta (\mathbf n_2) \, {\mathbb {I}}\big [ \mathbf n_1 + \mathbf n_2 \notin \mathcal {F}_{\{b\}}\big ]. \end{aligned}$$

To demonstrate (2.14), first notice that if $\mathbf n_1 + \mathbf n_2 \notin {\mathcal {F}}_B$, then there should be a connected component of $\mathbf n_1 + \mathbf n_2$ whose intersections with *A* and *B* are two sets $A'$ and $B'$ of odd cardinality, and furthermore this component should not contain $\mathfrak {g}$. The latter assumption is valid since if all such components contained $\mathfrak {g}$, then inevitably $\mathbf n_1 + \mathbf n_2 \in \mathcal {F}_B$. Therefore we have

2.15 $$\begin{aligned}&\sum _{\begin{array}{c} \partial \mathbf n_1 = A \cup B\\ \partial \mathbf n_2 = \emptyset \end{array}} \, \omega _\beta (\mathbf n_1) \, \omega _\beta (\mathbf n_2) \, {\mathbb {I}} [\mathbf n_1 + \mathbf n_2 \notin {\mathcal {F}}_B] \nonumber \\&\quad \le \sum _{\begin{array}{c} A' \subset A \\ | A'| \text { is odd } \end{array}} \sum _{\begin{array}{c} B' \subset B \\ | B'| \text { is odd } \end{array}} \sum _{\begin{array}{c} \partial \mathbf n_1 = A \cup B\\ \partial \mathbf n_2 = \emptyset \end{array}} \, \omega _\beta (\mathbf n_1) \, \omega _\beta (\mathbf n_2) \, {\mathbb {I}} \nonumber \\&\qquad [ \, {\mathcal {C}}_{\mathbf n_1 + \mathbf n_2} (A') \cap \big ( A \cup B \cup \{\mathfrak {g}\} \big )= A' \cup B' ]\,. \end{aligned}$$

We can write the third summation on the right hand side of the above display as

$$\begin{aligned}&\sum _{S \in \mathbf{S}} \, \sum _{\begin{array}{c} \, \, \mathbf n_1, \mathbf n_2 \in \mathbb {N}^{E_S} \\ \partial \mathbf n_1 = A'\\ \partial \mathbf n_2 = B' \end{array}} \, \omega _\beta (\mathbf n_1) \, \omega _\beta (\mathbf n_2) \, {\mathbb {I}} [ {\mathcal {C}}_{\mathbf n_1 + \mathbf n_2} (A') = S ]\\&\quad \times \Big ( \langle \sigma _{A \cup B {\setminus } (A' \cup B')} \rangle _{G{\setminus } S} \sum _{\begin{array}{c} \mathbf n_1, \mathbf n_2 \in \mathbb {N}^{E{\setminus } {E_S}} \\ \partial \mathbf n_1 =\emptyset \\ \partial \mathbf n_2 = \emptyset \end{array}} \, \omega _\beta (\mathbf n_1) \, \omega _\beta (\mathbf n_2) \Big ) \end{aligned}$$

where $\mathbf{S}$ is the set of all $S \subset V^+$ satisfying $S \cap ( A \cup B \cup \{\mathfrak {g}\} )= A' \cup B'$, and $E_S$ is the set of edges with at least one vertex in *S*. But this is bounded by

$$\begin{aligned} \sum _{\begin{array}{c} \partial \mathbf n_1 = A'\\ \partial \mathbf n_2 = B' \end{array}} \, \omega _\beta (\mathbf n_1) \, \omega _\beta (\mathbf n_2) \, {\mathbb {I}}[ \, \mathfrak {g} \notin {\mathcal {C}}_{\mathbf n_1+\mathbf {n}_2} (A') = {\mathcal {C}}_{\mathbf n_1+\mathbf {n}_2} (B') ]\,, \end{aligned}$$

plugging which into (2.15), we get

$$\begin{aligned}&\sum _{\begin{array}{c} \partial \mathbf n_1 = A \cup B\\ \partial \mathbf n_2 = \emptyset \end{array}} \, \omega _\beta (\mathbf n_1) \, \omega _\beta (\mathbf n_2) \, {\mathbb {I}} [\mathbf n_1 + \mathbf n_2 \notin {\mathcal {F}}_B] \nonumber \\&\quad \quad \le \sum _{\begin{array}{c} A' \subset A \\ | A'| \text { is odd } \end{array}} \sum _{\begin{array}{c} B' \subset B \\ | B'| \text { is odd } \end{array}} \sum _{\begin{array}{c} \partial \mathbf n_1 = A'\\ \partial \mathbf n_2 = B' \end{array}} \, \omega _\beta (\mathbf n_1) \, \omega _\beta (\mathbf n_2) \, {\mathbb {I}}[ \, \mathfrak {g} \notin {\mathcal {C}}_{\mathbf n_1+\mathbf {n}_2} (A') = {\mathcal {C}}_{\mathbf n_1+\mathbf {n}_2} (B') ]\,. \end{aligned}$$

Now, for every $A' \subset A$ and $B' \subset B$,

2.16 $$\begin{aligned}&\sum _{\begin{array}{c} \partial \mathbf n_1 = A' \cup B'\\ \partial \mathbf n_2 = \emptyset \end{array}} \, \omega _\beta (\mathbf n_1) \, \omega _\beta (\mathbf n_2) \, {\mathbb {I}}\big [ \mathfrak {g} \notin {\mathcal {C}}_{\mathbf n_1 + \mathbf n_2} (A') = {\mathcal {C}}_{\mathbf n_1 + \mathbf n_2} (B') \big ] \nonumber \\&\quad \le \sum _{\begin{array}{c} \partial \mathbf n_1 = A' \cup B'\\ \partial \mathbf n_2 = \emptyset \end{array}} \, \omega _\beta (\mathbf n_1) \, \omega _\beta (\mathbf n_2) \, {\mathbb {I}}\big [ \mathfrak {g} \notin {\mathcal {C}}_{\mathbf n_1 + \mathbf n_2} (A') \cup {\mathcal {C}}_{\mathbf n_1 + \mathbf n_2} (B') \big ] \nonumber \\&\quad \le \sum _{S \in \mathbf{S'}} \, \, \sum _{\begin{array}{c} \, \, \mathbf n_1, \mathbf n_2 \in \mathbb {N}^{ E_S} \\ \partial \mathbf n_1 = \emptyset \\ \partial \mathbf n_2 = \emptyset \end{array}} \, \omega _\beta (\mathbf n_1) \, \omega _\beta (\mathbf n_2) \, {\mathbb {I}}\big [ \mathcal {C}_{\mathbf n_1 +\mathbf n_2} (\mathfrak {g}) = S \big ] \, \langle \sigma _{A' \cup B'} \rangle _{G{\setminus } S} \nonumber \\&\qquad \sum _{\begin{array}{c} \mathbf n_1, \mathbf n_2 \in \mathbb {N}^{E{\setminus } {E_S}} \\ \partial \mathbf n_1 = \emptyset \\ \partial \mathbf n_2 = \emptyset \end{array}} \, \omega _\beta (\mathbf n_1) \, \omega _\beta (\mathbf n_2), \end{aligned}$$

where $\mathbf{S'}$ is the set of all $S \subset V^+$ such that $S \cap (A' \cup B' \cup \{ \mathfrak {g}\}) = \{ \mathfrak {g}\}$. Since $A'$ and $B'$ are sets of odd cardinality, one can use the correlation inequality

$$\begin{aligned} \langle \sigma _{A' \cup B'} \rangle _{G {\setminus } S} \le \sum _{\begin{array}{c} a \in A'\\ b \in B' \end{array}} \langle \sigma _{a} \sigma _{b} \rangle _{G {\setminus } S} \, \end{aligned}$$

coming from the Edwards–Sokal coupling and the fact that $\mathcal F_{A'\cup B'}$ is included in the event that some $a\in A'$ is connected to some $b\in B'$. Substituting the above inequality in (2.16) and resuming over $S\in \mathbf{S'}$ gives us

$$\begin{aligned} \sum _{\begin{array}{c} \partial \mathbf n_1 = A'\\ \partial \mathbf n_2 = B' \end{array}} \, \omega _\beta (\mathbf n_1) \, \omega _\beta (\mathbf n_2) \,&{\mathbb {I}}\big [ \mathfrak {g} \notin {\mathcal {C}}_{\mathbf n_1 + \mathbf n_2} (A') = \mathcal C_{\mathbf n_1 + \mathbf n_2} (B') \big ] \\&\le \sum _{\begin{array}{c} a \in A' \\ b \in B' \end{array}} \sum _{\begin{array}{c} \partial \mathbf n_1 = \{a, b\}\\ \partial \mathbf n_2 = \emptyset \end{array}} \, \omega _\beta (\mathbf n_1) \, \omega _\beta (\mathbf n_2) \, {\mathbb {I}}\big [ \mathfrak {g} \notin {\mathcal {C}}_{\mathbf n_1 + \mathbf n_2} (a) = {\mathcal {C}}_{\mathbf n_1 + \mathbf n_2} (b) \big ]. \end{aligned}$$

Summing over $A'$ and $B'$ gives us (2.14) and concludes the proof. $\quad \square$

*In the rest of the article*, *pand*$\beta$*are fixed so that*$1-p=e^{-2\beta }$*and dropped from the notation. *

## Applications of Pisztora’s Coarse-Grain Approach

The next subsection introduces the notion of good blocks. We then use a renormalization scheme to deduce that all big boxes are connected in FK-Ising. The last subsection derives Theorem 1.3 from Proposition 1.5.

### Blocks and good blocks

For $k\ge 1$ and a set *S*, introduce the set ${\mathcal {B}}_k(S)$ of boxes $\Lambda _{k}(x)\subset S$ with $x\in k\mathbb {Z}^d$. From now on, we call an element of ${\mathcal {B}}_k(S)$ a *block* and often identify it with the set of its edges. Call a block ${\mathbf {B}}$*good* in $\omega$ if

- $\omega _{|{\mathbf {B}}}$ contains a cluster touching all the 2*d* faces of ${\mathbf {B}}$;
- Any open path of length *k* in ${\mathbf {B}}$ is included in this cluster.

Boxes of this type were used by Pisztora to derive surface order large deviation estimates for the Ising, Potts and FK-percolation models. While the notion of good box there is slightly different, we refer to the definition of *U* before [Pis96, Theorem 3.1] for a stronger notion than the notion above. The papers [Pis96] and [Bod05] together imply, for every $p>p_c$, the existence of $c>0$ such that for every *k* and every boundary condition $\xi$,

3.1 $$\begin{aligned} \phi ^\xi _{\Lambda _{2k}}[\Lambda _{k} \text { is good}] > 1 - \mathrm {e}^{-ck}. \end{aligned}$$

#### Lemma 3.1

For every $p>p_c$, there exists $c>0$ such that for every $n\le N\le e^{n^\alpha }$ (with $\alpha <d-1$) and every $x,y\in \mathbb {Z}^d$ such that $\Lambda _n(x),\Lambda _n(y)\subset \Lambda _N$,

3.2 $$\begin{aligned} \phi ^0_{\Lambda _N}[ \Lambda _n(x) \longleftrightarrow \Lambda _n(y)] \ge 1- \exp (-c n ^{d-1}). \end{aligned}$$

#### Proof

Fix $p<1$. Choose $\varepsilon >0$ small enough (see later) and *k* large enough that

$$\begin{aligned} \phi ^\xi _{\Lambda _{2k} }[\Lambda _{k} \text { is good}] > 1 - \varepsilon \end{aligned}$$

for every boundary condition $\xi$. Define a site percolation $\eta$ on ${\mathcal {B}}_k(\Lambda _N)$ by saying that ${\mathbf {B}}$ is open if it is good, and closed otherwise. For a box ${\mathbf {B}}$, define $M(\mathbf B)$ to be the set of all the boxes in ${\mathcal {B}}_k(\Lambda _N)$ whose centers are at a $\ell ^\infty$ distance at most 3*k* of the center of $\mathbf{B}$. Note that,

$$\begin{aligned} \phi ^{0}_{\Lambda _N} [ \eta _{\,{\mathbf {B}}} \, \vert \, \eta _{\vert _{M({\mathbf {B}})^c}} ] \ge 1 -\varepsilon . \end{aligned}$$

One deduces from [LSS97] that the process $\eta$ dominates a Bernoulli percolation ${\tilde{\eta }}$ with parameter *p* provided that $\varepsilon$ is small enough.

A result of Deuschel and Pisztora [DP96] shows that for *p* close enough to 1 and $n\ge 1$, the probability that, for a fixed box $\Lambda _n(x)$, there exists an open cluster in ${\tilde{\eta }}$ containing more than three fourths of the blocks in ${\mathcal {B}}_k(\Lambda _n(x))$ is larger than $1-\exp [-2cn^{d-1}]$. The domination of ${\tilde{\eta }}$ by $\eta$ together with a union bound shows that this cluster also exists in $\eta$. Therefore, with probability

$$\begin{aligned} 1-|\Lambda _N|\exp (-2cn^{d-1})\ge 1-|\Lambda _{\exp (n^\alpha )}|\exp (-2cn^{d-1})\ge 1-\exp (-cn^{d-1}) \end{aligned}$$

(for *n* large enough), every box $\Lambda _n(x)$ in $\Lambda _N$ satisfies that there exists an open cluster in $\eta$ containing more than half the blocks contained in ${\mathcal {B}}_k(\Lambda _n(x))$.

As a immediate consequence, on this event, all these clusters [meaning for every $\Lambda _n(x)$] must in fact be the same cluster. In particular, there exist paths of pairwise neighboring good blocks going between any two boxes of size *n* in $\Lambda _N$. Property (a) of a good block implies that there must be an open path of length at least *k* in the common region between two neighboring good blocks. Property (b), on the other hand, implies that such a path must belong to the largest clusters of each of these blocks, i.e. the largest clusters of these blocks are connected. Together these give us that there exists an open path in $\omega$ between any two boxes of size *n*. $\quad \square$

#### Remark 3.2

The result of [LSS97] is quantitative. Since the probability of being good is larger than $1-\mathrm {e}^{-ck}$, the process $\eta$ dominates a Bernoulli percolation of parameter *p* equal to $1-\mathrm {e}^{-c'k}$ for some $c'=c'(c)>0$ independent of *k*.

### From Proposition 1.5 to Theorem 1.3

We now prove Theorem 1.3 using Proposition 1.5. Fix some boundary conditions $\xi$. We construct a coupling $\Phi ^{\xi ,1}_{\Lambda _n,}$ on pairs of configurations $(\omega ^\xi ,\omega ^1)$ with $\phi ^\xi _{\Lambda _n}$ being the law of the first marginal and $\phi ^1_{\Lambda _n}$ the one of the second marginal. Call a block ${\mathbf {B}}\in \mathcal B_k(\Lambda _n)$*very good* in $(\omega ^\xi ,\omega ^1)$ if it is good in $\omega ^\xi _{|{\mathbf {B}}}$ and $\omega ^\xi _{|\mathbf B}=\omega ^1_{|{\mathbf {B}}}$.

We construct algorithmically a coupling $\Phi ^{\xi ,1}_{\Lambda _n}$ block by block following steps indexed by *t*. We assume that *k* divides *n* (the construction can be adapted in a trivial fashion to the general case). Below, $C_t$ will denote the set of edges *e* for which $(\omega ^\xi _e,\omega ^1_e)$ is sampled before step *t*. The set $A_t$ is the set of blocks that have been sampled up to time *t*. Set

$$\begin{aligned} {\left\{ \begin{array}{ll}A_0:=\emptyset ,\\ B _ 0:=\{{\mathbf {B}}\in {\mathcal {B}}_k(\Lambda _n),\mathbf B\cap (\Lambda _{n}{\setminus }\Lambda _{n-1})\ne \emptyset \} ,\\ C_0 := \emptyset .\end{array}\right. } \end{aligned}$$

(The set $B_0$ corresponds to blocks adjacent to the boundary of $\Lambda _n$.) At Step *t*, the algorithm proceeds as follows:

- If $B_t=\emptyset$, sample $\omega ^\xi _{|E_n{\setminus } C_{t}} = \omega ^1_{|E_n{\setminus } C_{t}}$ according to the measure $\phi ^\xi _{\Lambda _n} ( \, . \, \vert \omega ^\xi _{|C_{t}})$ and terminate the algorithm.
- If $B_t\ne \emptyset$, choose a block ${\mathbf {B}}\in B_{t}$ and define $D_t :={\mathbf {B}} {\setminus } C_t$. Then, sample $\omega ^\xi _{|D_t}\le \omega ^1 _{|D_t}$ such that $\omega ^\xi _{|D_t}$ has the law $\phi ^\xi _{\Lambda _n} [\cdot _{|D_t} \vert \omega ^\xi _{|C_t}]$ and $\omega ^1_{|D_t}$ the law $\phi ^1_{\Lambda _n} [ \cdot _{|D_t} \vert \omega ^1_{|C_t}]$. Finally, if $N({\mathbf {B}})$ denotes the set of blocks in $\mathcal B_k(\Lambda _n)$ that intersect ${\mathbf {B}}$, set
  $$\begin{aligned} {\left\{ \begin{array}{ll} A_{t+1} := A_t \cup \{{\mathbf {B}}\},\\ B_{t+1} := B_t {\setminus } \{{\mathbf {B}}\}\text { if }{\mathbf {B}}\text { is very good and }B_t\cup N({\mathbf {B}}){\setminus } A_{t+1} \text { otherwise,}\\ C_{t+1} := C_t \cup D_t. \end{array}\right. } \end{aligned}$$

When the algorithm terminates, set *T* for the terminal time and return $(\omega ^\xi ,\omega ^1)$.

#### Lemma 3.3

The measure $\Phi ^{\xi ,1}_{\Lambda _n}$ satisfies that

- $\omega ^\xi$ has law $\phi ^\xi _{\Lambda _n}$ and $\omega ^1$ has law $\phi ^1_{\Lambda _n}$,
- $$\omega ^\xi \le \omega ^1$$,
- $$\omega ^\xi _e=\omega ^1_e$$ if $$e\notin C_{T}$$.

#### Proof

To prove this lemma, it suffices to prove that the boundary conditions induced on $E_n{\setminus } C_T$ by $\omega ^\xi _{|C_T}$ and $\omega ^1_{|C_T}$ are the same. Indeed, the three items follow readily from this observation and the joint spatial Markov property. Since $\omega ^\xi _{|C_T}\le \omega ^1_{|C_T}$, it suffices to prove that for every vertices *x* and *y* on the boundary of $E_n{\setminus } C_T$,

3.3 $$\begin{aligned} x{\mathop {\longleftrightarrow }\limits ^{\omega ^1_{|C_T}}}y\ \ \Longrightarrow \ \ x{\mathop {\longleftrightarrow }\limits ^{\omega ^\xi _{|C_T}}}y\quad . \end{aligned}$$

If $C_T=E_n$ there is nothing to prove. If $C_T\ne E_n$, let

$$\begin{aligned} Y:={\mathcal {B}}_k(\Lambda _n){\setminus } A_T\qquad \text { and }\qquad Z:=\{{\mathbf {B}}\in A_T\text { intersecting a block in }Y\}, \end{aligned}$$

where below we also identify *Z* with the set of edges in its blocks. The fact that $B_T= \emptyset$ implies that $\omega ^\xi$ and $\omega ^1$ coincide on *Z* and every ${\mathbf {B}}\in Z$ is very good for $\omega ^1$ and $\omega ^\xi$. Now, for every connected component *C* of $C_T$, the set of blocks in *Z* that intersect *C* are connected in the following sense: every two such blocks $\mathbf{B}$ and $\mathbf{B'}$ are connected by a sequence of blocks $\mathbf{B}=\mathbf{B}_1,\dots ,\mathbf{B}_s=\mathbf{B'}$ of *Z* such that $\mathbf{B}_i\cap \mathbf{B}_{i+1}\ne \emptyset$ for every $1\le i<s$. The discussion in the proof of Lemma 3.1 implies that all the big clusters in these blocks are connected to each other inside *Z*. Thus, if two vertices *x* and *y* on the boundary of $C_T$ are connected in $\omega ^\xi _{|C_T}$, then they already are in $\omega ^\xi _{|Z}=\omega ^1_{|Z}$. This proves (3.3) and therefore concludes the proof. $\quad \square$

#### Proof of Theorem 1.3

Let *D* be the number of blocks in $M(\mathbf{B})$ (recall the definition of $M(\mathbf{B})$ from the proof of Lemma 3.1 and observe that the choice of $\mathbf{B}$ is irrelevant here), and choose $\varepsilon$ so that $2D\varepsilon <1/e$. By Proposition 1.5, pick *k* large enough that

$$\begin{aligned} \sum _{e\in \Lambda _{k}}\phi ^1_{\Lambda _{2k}}[\omega _e]-\phi ^0_{\Lambda _{2k}}[\omega _e]\le |\Lambda _{k}|e^{-c(\log k)^{1+c}}\le \varepsilon . \end{aligned}$$

Also, assume that *k* is chosen large enough that the probability of being good is larger than $1-\varepsilon$ [this is doable by using (3.1)].

By the Markov property (2.1), (1.7) follows from the next claim: for every boundary condition $\xi$ on $\mathbb {Z}^d$ and every event *A* depending on edges in $E_{n/2}$ only,

3.4 $$\begin{aligned} |\phi ^\xi _{\Lambda _n}[A]-\phi ^1_{\Lambda _n}[A]|\le \Phi ^{\xi ,1}[C_T\cap E_{n/2}\ne \emptyset ]\le \exp [-cn]. \end{aligned}$$

The first inequality follows directly from the third item of Lemma 3.3. We therefore focus on the second one. Assume that $C_T\cap E_{n/2}\ne \emptyset$. Then, there must exist a sequence of times $t_1<t_2<\dots <t_s$ with $s\ge n/(16k)$ such that the blocks ${\mathbf {B}}_1,\dots ,{\mathbf {B}}_s$ used by the algorithm constructing $\Phi ^{\xi ,1}_{\Lambda _n}$ at times $t_1,\dots ,t_s$ are disjoint, not very good, and such that $\mathbf{B}_{i+1}\in M(\mathbf{B}_i)$ for every $1\le i<s$. The choice of *k* immediately implies that conditioned on $\omega ^\xi$ and $\omega ^1$ in the blocks ${\mathbf {B}}_1,\dots ,{\mathbf {B}}_i$, the block ${\mathbf {B}}_{i+1}$ is not very good with probability smaller than or equal to $2\varepsilon$. We deduce that

3.5 $$\begin{aligned} \Phi ^{\xi ,1}_{\Lambda _n} [ C_T\cap E_{n/2}\ne \emptyset ] \le (D2\varepsilon )^{n/(16k)}. \end{aligned}$$

The choice of $\varepsilon$ proves (3.4) with $c=1/(16k)$. $\quad \square$

## Proof of Proposition 1.5

The proof of Proposition 1.5 can be decomposed into several steps. We first prove a very weak mixing property (where the Radon–Nikodym derivative is bounded from above by $\exp (cn^{d-1})$. We then use this property to show Proposition 1.5, but basing our study on two technical lemmata whose proofs are postponed to the next section.

### Connection probability between boxes for double random current

The main result of this section is the proposition below. The proof relies on a number of properties of the random current, combined with a mixing property for the Fortuin–Kasteleyn percolation.

#### Proposition 4.1

Fix $\beta >\beta _c$. There exists $c>0$ such that for every $N \ge 2n$, every $x, y,w,z \in \Lambda _{N}$ with $w, z \notin \Lambda _n(x) \cup \Lambda _n(y)\subset \Lambda _N$, we have

In the whole section, we will use the following notation. Without loss of generality, we can assume that *n* is even. Assume that $\Lambda _n(x)\cap \Lambda _n(y)=\emptyset$. Construct the graphs $\Lambda _N^{\bullet \bullet }:=\Lambda _N^{\bullet \bullet }(x,y,n)$ and $(\Lambda _N^{\bullet \bullet })^+:=(\Lambda _N^{\bullet \bullet }(x,y,n))^+$ obtained from $\Lambda _N$ and $\Lambda _N^+$ by replacing $\Lambda _{n/2}(x)$ and $\Lambda _{n/2}(y)$ by two vertices $\mathbf{x}$ and $\mathbf{y}$, which are connected to each vertex outside $\Lambda _{n/2}(x)\cup \Lambda _{n/2}(y)$ by the number of edges between this vertex and $\Lambda _{n/2}(x)$ (resp. $\Lambda _{n/2}(y)$). Similarly, one defines $\Lambda _{n}^\bullet (x)$ to be the graph obtained from $\Lambda _{n}(x)$ by merging all the vertices in $\Lambda _{{n/2}}(x)$ following the same procedure as for $\Lambda _N^{\bullet \bullet }$. Note that the FK-Ising and Ising models on those graphs can be seen as models on the original graphs, for which edges *e* in $\Lambda _{n/2}(x) \cup \Lambda _{n/2}(y)$ have $p_e=1$ (for the FK-Ising) and have infinite coupling constants (for the Ising model). This observation is useful to keep in mind when applying (2.8) for instance.

#### Proof

If $\Lambda _n(x)\cap \Lambda _n(y)\ne \emptyset$, one does not need to do anything. We therefore assume that $\Lambda _n(x)\cap \Lambda _n(y)=\emptyset$ and consider the graph $\Lambda _N^{\bullet \bullet }$ defined above. Equation (2.12) implies that4.1where in the second inequality we used Griffiths’ inequalities (2.7) and (2.8). The Edwards–Sokal coupling (2.5) and the FKG inequality (2.3) (the measure on $\Lambda _N^{\bullet \bullet }$ can be understood as the measure on $\Lambda _N$ with edges in $\Lambda _{n/2}(x)\cup \Lambda _{n/2}(y)$ conditioned to be open) imply that

4.2 $$\begin{aligned} \langle \sigma _{\mathbf{x}}\sigma _{\mathbf{y}} \rangle _{\Lambda _N^{\bullet \bullet }}=\phi _{\Lambda _N^{\bullet \bullet }}[\mathbf{x}\leftrightarrow \mathbf{y}]\ge \phi _{\Lambda _N}[\Lambda _{{n/2}}(x)\leftrightarrow \Lambda _{{n/2}}(y)]\ge 1-\exp (-cn^{d-1}), \end{aligned}$$

where the last inequality follows from Lemma 3.1.

Assume for a moment that for every $\varepsilon >0$, one can choose *n* large enough that for every choice of *N* and *x*, *y*, for every event ${\mathcal {E}}$ depending on $(\mathbf {n}_1,\mathbf {n}_2)$ on edges in $E:=E_N{\setminus } (E_{n}(x)\cup E_{n}(y))$ only, we have that

4.3 $$\begin{aligned} {\mathbb {P}}^{\{w, z\}, \emptyset }_{\Lambda _N^+, \Lambda _N}[{\mathcal {E}}]\le e^{\varepsilon n^{d-1}}{\mathbb {P}}^{\{w, z\}, \emptyset }_{(\Lambda _N^{\bullet \bullet })^+, \Lambda _N^{\bullet \bullet }}[{\mathcal {E}}]. \end{aligned}$$

Then, one would deduce from plugging the estimate (4.2) into (4.1), and then using (4.3) (together with the fact that if $\mathbf{x}$ is connected to $\mathbf{y}$, then $\Lambda _n(x)$ is connected to $\Lambda _n(y)$), thatwhich would conclude the proof. We therefore focus on proving (4.3). Let $\Lambda :=\Lambda _n(x) \cup \Lambda _n(y)$ and consider a current $\mathbf{m}\in \mathbb {N}^E$ with $\partial \mathbf{m}\subset \partial \Lambda$. Let us also consider the event $\mathcal E_\mathbf{m}$ that $\mathbf {n}$ is equal to $\mathbf{m}$ on *E*. A trivial manipulation using the expression of weights for currents (in the first line) and then Griffiths inequality (2.8) in the second give that

$$\begin{aligned} \frac{{\mathbb {P}}^{\emptyset }_{\Lambda _N}[{\mathcal {E}}_\mathbf{m}]}{{\mathbb {P}}^{\emptyset }_{\Lambda _N^{\bullet \bullet }}[{\mathcal {E}}_\mathbf{m}]}&=\frac{\langle \sigma _{\partial \mathbf{m}} \rangle _{\Lambda _{n}(x)\cup \Lambda _{n}(y)}}{\langle \sigma _{\partial \mathbf{m}} \rangle _{\Lambda _{n}^\bullet (x)\cup \Lambda _{n}^\bullet (y)}}\,\frac{\displaystyle \sum _{\mathbf m' \in \mathbb {N}^E, \, \partial \mathbf m'\subset \partial \Lambda }w_\beta (\mathbf m')\langle \sigma _{\partial \mathbf m'} \rangle _{\Lambda _{n}^\bullet (x)\cup \Lambda _{n}^\bullet (y)}}{\displaystyle \sum _{\mathbf m' \in \mathbb {N}^E, \, \partial \mathbf m'\subset \partial \Lambda }w_\beta (\mathbf m')\langle \sigma _{\partial \mathbf m'} \rangle _{\Lambda _{n}(x)\cup \Lambda _{n}(y)}}\\&\le \max _{\begin{array}{c} A\subset \partial \Lambda _{n}(x)\\ |A|\text { even} \end{array}}\frac{\langle \sigma _A \rangle _{\Lambda _{n}^\bullet (x)}}{\langle \sigma _A \rangle _{\Lambda _{n}(x)}}\times \max _{\begin{array}{c} B\subset \partial \Lambda _{n}(y)\\ |B|\text { even} \end{array}}\frac{\langle \sigma _B \rangle _{\Lambda _{n}^\bullet (y)}}{\langle \sigma _B \rangle _{\Lambda _{n}(y)}}, \end{aligned}$$

where the sums on the first line are on currents on *E* (with the right sources). A similar identity can be derived for ${\mathbb {P}}^{\{z,w\}}_{\Lambda _N^+}$ and ${\mathbb {P}}^{\{z,w\}}_{(\Lambda _N^{\bullet \bullet })^+}$ instead of ${\mathbb {P}}^{\emptyset }_{\Lambda _N}$ and ${\mathbb {P}}^{\emptyset }_{\Lambda _N^{\bullet \bullet }}$.

We deduce, by decomposing on possible values of $(\mathbf {n}_1,\mathbf {n}_2)$ on *E*, that

$$\begin{aligned} {\mathbb {P}}^{\{w, z\}, \emptyset }_{\Lambda _N^+, \Lambda _N}[{\mathcal {E}}]\le \Big (\max _{\begin{array}{c} A\subset \partial \Lambda _{n}\\ |A|\text { even} \end{array}}\frac{\langle \sigma _A \rangle _{\Lambda _{n}^\bullet }}{\langle \sigma _A \rangle _{\Lambda _{n}}}\Big )^4\ {\mathbb {P}}^{\{w, z\}, \emptyset }_{(\Lambda _N^{\bullet \bullet })^+, \Lambda _N^{\bullet \bullet }}[{\mathcal {E}}], \end{aligned}$$

so that (4.3) follows from the following lemma. $\quad \square$

#### Lemma 4.2

Fix $\beta >\beta _c$ and $\varepsilon >0$. Then, for *n* large enough, we have that for every $A\subset \partial \Lambda _n$,

4.4 $$\begin{aligned} \langle \sigma _A \rangle _{\Lambda _n^\bullet } \le \mathrm {e}^{\varepsilon n^{d-1}}\langle \sigma _A \rangle _{\Lambda _n}. \end{aligned}$$

#### Proof

Fix $\beta >\beta _c$ and set $p=1-e^{-2\beta }>p_c$. By (2.5), we must prove that $\phi _{\Lambda _n^\bullet }^0[{\mathcal {F}}_A]\le \mathrm {e}^{\varepsilon n^{d-1}}\phi _{\Lambda _n}^0[{\mathcal {F}}_A]$. Note that since $\varepsilon$ is arbitrary and since ${\mathcal {F}}_A$ occurs if all the edges on the boundary of $\Lambda _n$ are open (and therefore has a probability larger than $(\tfrac{p}{2-p})^{d|\partial \Lambda _n|}$), it suffices to show that

4.5 $$\begin{aligned} \phi _{\Lambda _n^\bullet }^0[{\mathcal {F}}_A]\le \mathrm {e}^{-cn^d}+n\,\big (\tfrac{2-p}{p}\big )^{\varepsilon n^{d-1}}\phi _{\Lambda _n}^0[{\mathcal {F}}_A]. \end{aligned}$$

We do this by constructing a coupling $\Phi$ on pairs $(\omega ,\omega ^\bullet )$ of percolation configurations on $\{0,1\}^{E_n}$ for which $\omega \le \omega ^\bullet$ have respective laws $\phi _{\Lambda _n}^0$ and $\phi _{\Lambda _n^\bullet }^0$ (for consistency, we see a configuration on $\Lambda _n^\bullet$ as a configuration on $E_n$ for which edges in $E_{n/2}$ are automatically open).

**Fig. 1:**
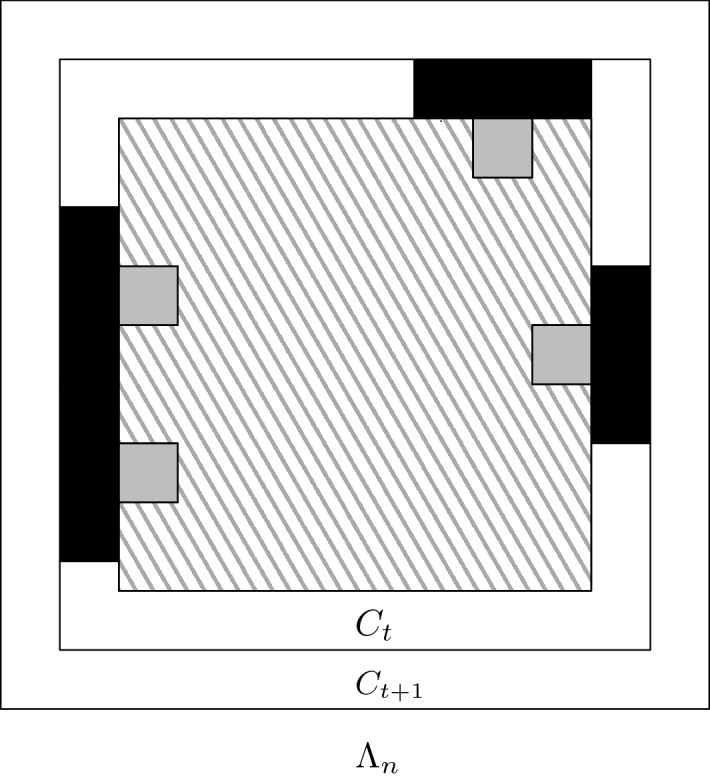
**A realization of the event **${\mathcal {H}}_t$. The grey boxes are the bad blocks in $C_t$ that are adjacent to $C_{t + 1} {\setminus } C_t$. The dark rectangles represent the edges in $D_t$

Consider *k* to be fixed later and assume that *k* divides *n* / 2 (one can trivially adapt the proof if *k* does not). Also, set $T:=n/(4k)$. A block ${\mathbf {B}}$ is called *bad* if it is either not good or if $\omega _{|{\mathbf {B}}}\ne \omega ^\bullet _{|{\mathbf {B}}}$. For every $t\in [T,2T]$, define the three sets $C_t:=E_{2k t}$, $D_t$ the set of edges in $C_{t + 1}{\setminus } C_t$ within a distance of 2*k* of a bad block included in $C_t$, and $D'_t:=C_{t+1}{\setminus } (C_t\cup D_t)$; see Fig. 1. Note that $C_t$ is a deterministic set, but $D_t$ and $D'_t$ are random variables which are measurable in terms of $(\omega _{|C_t},\omega ^\bullet _{|C_t})$.

Then, the coupling is constructed as follows. First, sample $\omega _{|E_{n/2}}$ according to $\phi _{\Lambda _n}(\cdot _{|E_{n/2}})$ (recall that the edges of $\omega ^\bullet$ are necessarily open in $E_{n/2}$). Then, for every $t\ge T$,

- Sample $\omega _{|D_t}\le \omega ^\bullet _{|D_t}$ according to $\phi _{\Lambda _n}^0(\cdot _{|D_t}|\omega _{|C_t})$ and $\phi ^0_{\Lambda _n^\bullet }(\cdot _{|D_t}|\omega ^\bullet _{|C_t})$,
- Sample $\omega _{|D'_t}\le \omega ^\bullet _{|D'_t}$ according to $\phi _{\Lambda _n}^0(\cdot _{|D'_t}|\omega _{|C_t\cup D_t})$ and $\phi ^0_{\Lambda _n^\bullet }(\cdot _{|D'_t}|\omega ^\bullet _{|C_t\cup D_t})$.

For $t\in [T,2T]$, we call ${\mathcal {G}}_t$ the event that there are fewer than $\varepsilon n^{d-1}$ edges in $D_t$. The inequality (4.5) follows readily from the following two claims. $\quad \square$

#### Claim 1

For every $t\ge T$, we have that

$$\begin{aligned} \Phi [\{\omega ^\bullet \in {\mathcal {F}}_A\}\cap {\mathcal {G}}_t]\le \big (\tfrac{2-p}{p}\big )^{\varepsilon n^{d-1}}\Phi [\omega \in {\mathcal {F}}_A]. \end{aligned}$$

#### Claim 2

There exist *k* and $c>0$ such that for every *n* large enough,

$$\begin{aligned} \Phi \big [\bigcap _{t\ge T}{\mathcal {G}}_t^c\big ]\le \mathrm {e}^{-cn^d}. \end{aligned}$$

To conclude, we only need to prove those claims.

#### Proof of Claim 1

Let ${\mathcal {H}}_t$ be the event that every edge in $D_t$ is open. We refer the reader to Fig. 1 for an illustration. On ${\mathcal {H}}_t\cap {\mathcal {G}}_t$, the boundary conditions on $E_n{\setminus }(C_t\cup D_t)$ induced by $\omega$ and $\omega ^\bullet$ are the same. Since ${\mathcal {H}}_t\cap {\mathcal {G}}_t$ depends on edges in $C_t\cup D_t$ only, the joint Markov property of the coupling implies that $\omega _{|E_n{\setminus } (C_t\cup D_t)}=\omega ^\bullet _{|E_n{\setminus } (C_t\cup D_t)}$ and $\omega ^\bullet \in {\mathcal {F}}_A$ if and only if $\omega \in \mathcal F_A$. We deduce that

4.6 $$\begin{aligned} \Phi [\{\omega ^\bullet \in {\mathcal {F}}_A\}\cap {\mathcal {H}}_t\cap \mathcal G_t]=\Phi [\{\omega \in {\mathcal {F}}_A\}\cap {\mathcal {H}}_t\cap \mathcal G_t]\le \Phi [\omega \in {\mathcal {F}}_A]. \end{aligned}$$

Conditioned on $(\omega ,\omega ^\bullet )_{|C_t}$, the joint FKG inequality and the joint Markov property imply that

4.7 $$\begin{aligned} \Phi [\{\omega ^\bullet \in {\mathcal {F}}_A\}\cap {\mathcal {H}}_t|(\omega ,\omega ^\bullet )_{|C_t}]\ge \Phi [\{\omega ^\bullet \in \mathcal F_A\}|(\omega ,\omega ^\bullet )_{|C_t}]\Phi [\mathcal H_t|(\omega ,\omega ^\bullet )_{|C_t}]. \end{aligned}$$

If $(\omega ,\omega ^\bullet )_{|C_t}\in {\mathcal {G}}_t$, at most $\varepsilon n^{d-1}$ edges must be open for ${\mathcal {H}}_t$ to occur. We deduce that

$$\begin{aligned} \Phi [{\mathcal {H}}_t|(\omega ,\omega ^\bullet )_{|C_t}]\ge \big (\tfrac{p}{2-p}\big )^{\varepsilon n^{d-1}}. \end{aligned}$$

Since ${\mathcal {G}}_t$ depends on edges in $C_t$ only, we may insert the previous estimate in (4.7) and then integrate on $(\omega ,\omega ^\bullet )_{|C_t}\in {\mathcal {G}}_t$ to obtain that

$$\begin{aligned} \Phi [\{\omega ^\bullet \in {\mathcal {F}}_A\}\cap {\mathcal {H}}_t\cap {\mathcal {G}}_t]\ge \Phi [\{\omega ^\bullet \in {\mathcal {F}}_A\}\cap {\mathcal {G}}_t] \big (\tfrac{p}{2-p}\big )^{\varepsilon n^{d-1}}. \end{aligned}$$

Putting this inequality into (4.6) concludes the proof of Claim 1.

#### Proof of Claim 2

For no ${\mathcal {G}}_t$ to occur, there must be at least $\varepsilon n^{d-1}/|E_{3k}|\times n/(4k)=:\varepsilon _k|E_n|$ bad blocks. As a consequence, one of the following three things must happen:

- There are more than $(\varepsilon _k/2)|E_n|$ blocks in $\mathcal B_k(\Lambda _n)$ that are not good.
- The number of open edges in $\omega ^\bullet _{|E_n{\setminus } E_{n/2}}$ is larger than $(\phi [\omega _e]+\varepsilon _k/4)|E_n{\setminus } E_{n/2}|$.
- The number of open edges in $\omega _{|E_n{\setminus } E_{n/2}}$ is smaller than $(\phi [\omega _e]-\varepsilon _k/4)|E_n{\setminus } E_{n/2}|$.

Note that these three events are involving either $\omega$ or $\omega ^\bullet$, so that we can now ignore the coupling $\Phi$. We bound the probability of each one of these events separately.

For the first item, Remark 3.2 enables us to choose *k* large enough that the process of good boxes dominates a Bernoulli percolation of parameter $p>1-\varepsilon _k$. We deduce from large deviations for iid Bernoulli variables that the probability of the event decays as $e^{-cn^d}$ uniformly in *n*, where $c=c(k,\varepsilon )>0$.

For the second item, the uniqueness of the infinite-volume measure $\phi$ implies that one may choose $K=K(k,\varepsilon )$ large enough that

$$\begin{aligned} \phi ^1_{\Lambda _K}\Big [\sum _{e\in E_K}\omega _e\Big ]< (\phi [\omega _e]+\varepsilon _k/4)|E_K|. \end{aligned}$$

Now, consider a family of balls of size *K* covering $\Lambda _n$ and with disjoint interiors. By sampling the FK-Ising measure $\phi ^1_{\Lambda _K}$ ball by ball (here, the order in which the balls are sampled is irrelevant), the comparison between boundary conditions (2.4) enables us to compare the number of open edges in $E_n{\setminus } E_{n/2}$ to a sum of independent random variables. The theory of large deviations implies that the probability of the second event is also bounded by $\exp (-cn^d)$ uniformly in *n*, where $c=c(K,k,\varepsilon )>0$.

The third item follows from the same reasoning as the second one (except it does not involve the uniqueness of the infinite-volume measure). $\square$

### Proof of proposition 1.5

In the rest of the paper, for a current $\mathbf n$ on $\Lambda _N$ or $\Lambda _N^+$, we use ${\mathcal {C}}_{\mathbf n}(S)$ to denote the set of all vertices *in*$\Lambda _N$ which are connected to *S* by $\mathbf n$. Notice that this is slightly different from the convention in Lemma 1.2 where we also included ${\mathfrak {g}}$ in $\mathcal C_{\mathbf n}(S)$.

From now on, we fix $N=N(n): = e ^{n^ \alpha }$ where $1<\alpha < d-1$, and $e =\{x,y\}$. Using (2.5) and a simple relation between the probability of $\omega _e=1$ and the probability that *x* and *y* are connected together, we find that4.8Notice that *x* and *y* are the sources of $\mathbf n_1$ and as such they must be connected to each other by $\mathbf n_1$ in $\Lambda _N^+$. Thus, when *x* is not connected to *y* in $\Lambda _N$, there must be a path between *x* and $\partial \Lambda _N$ in ${\mathcal {C}}_{\mathbf n_1}(x)$ on one side, and between *y* and $\partial \Lambda _N$ in ${\mathcal {C}}_{\mathbf n_1}(y)$ on the other side. Since $\{x, y\} \in E_{N/4}$, the path emanating from *y* must intersect at least $\tfrac{1}{2}N/n$ blocks in ${\mathcal {B}}_n(\Lambda _{N})$. Among these blocks, there is always either $\tfrac{1}{4}N/n$ many intersecting ${\mathcal {C}}_{\mathbf n_1 + \mathbf n_2}(x)$ or $\tfrac{1}{4}N/n$ many not intersecting it. Repeating a similar argument for the path between *x* and $\partial \Lambda _N$, we obtain that if *x* is not connected to *y* in $\mathbf {n}_1+\mathbf {n}_2$ and we define the following events,

$$\begin{aligned} {\mathcal {C}}_{x,y} :=&\big \{\text{ there } \text{ exist } \tfrac{1}{4}N/n \text{ blocks } \mathbf{B}\in {\mathcal {B}}_n(\Lambda _{N}) \text{ such } \text{ that } {\mathcal {C}}_{\mathbf n_1 + \mathbf n_2}(x) \cap \mathbf{B} = \emptyset \text{ and } {\mathcal {C}}_{\mathbf n_1}(y) \cap \mathbf{B}\\&\text{ contains } \text{ a } \text{ vertex } \text{ having } \text{ at } \text{ least } \text{ two } \text{ neighbors } \text{ in } {\mathcal {C}}_{\mathbf n_1}(y)\big \},\\ {\mathcal {B}}_{x,y} :=&\{\text{ there } \text{ exist } \tfrac{1}{4}N/n \text{ blocks } \mathbf{B}\in {\mathcal {B}}_n(\Lambda _{N}) \text{ such } \text{ that } \mathcal C_{\mathbf n_1 + \mathbf n_2}(x) \cap \mathbf{B} \ne \emptyset \text{ and } {\mathcal {C}}_{\mathbf n_1}(y) \cap \mathbf{B}\\&\text{ contains } \text{ a } \text{ vertex } \text{ having } \text{ at } \text{ least } \text{ two } \text{ neighbors } \text{ in } {\mathcal {C}}_{\mathbf n_1}(y)\}, \end{aligned}$$

then, either ${\mathcal {B}}_{x,y}$, ${\mathcal {B}}_{y,x}$ or ${\mathcal {C}} := {\mathcal {C}}_{x, y} \cap {\mathcal {C}}_{y, x}$ must occur.

Therefore, it suffices to show that the intersection of the event on the right hand side of (4.8) with either $\mathcal C$, ${\mathcal {B}}_{x, y}$ or ${\mathcal {B}}_{y,x}$ has very small probability. This is the subject of the two lemmata below which are proved in the next section.

#### Lemma 4.3

There exists $c>0$ such that for every *n* large enough such that $\{x,y\}\in E_{N/4}$,

#### Lemma 4.4

There exists $c>0$ such that for every *n* large enough such that $\{x,y\}\in E_{N/4}$,

Note that the bounds given below imply Proposition 1.5 since $N=\mathrm{e} ^{n^ \alpha }$ gives that the bounds on the right are of the form $\exp [-(\log N)^{(d-1)/\alpha }]$, and that $\alpha <d-1$.

## Proofs of lemmata 4.3 and 4.4

In the remainder of the paper, we will use *C* and *c* to denote finite, positive constants depending on at most $\beta$ and *d*. The values of these constants may vary from one line to the next. It might be helpful to think of *C* and *c* as large and small positive numbers respectively.

In order to prove these lemmata, we will use a *multi-valued map principle*.

### Lemma 5.1

Consider a probability space $({\mathcal {S}}, {\mathfrak {P}}({\mathcal {S}}), \mu )$ where ${\mathcal {S}}$ is at most countable and $\mathfrak P({\mathcal {S}})$ is the set of all subsets of ${\mathcal {S}}$ . Let $A, B \subset {\mathcal {S}}$ and ${\mathcal {R}} \subset A \times B$ be a relation satisfying the following two properties:

(i) $|{\mathcal {R}}(s)| \ge K$ for every $s \in A$ where ${\mathcal {R}}(s) := \{s' \in B:(s, s') \in {\mathcal {R}} \}$.
(ii) $\sum _{s \in {\mathcal {R}}^{-1}(s')} \mu (s)\le k \mu (s')$ for every $s' \in B$ where ${\mathcal {R}}^{-1}(s') := \{s \in A: (s,s') \in {\mathcal {R}} \}$.

Then, we have that

$$\begin{aligned} \mu (A) \le \frac{k}{K}\mu (B)\,. \end{aligned}$$

### Proof

This is a simple application of “counting in two ways”:

$$\begin{aligned} K\mu (A) \overset{(i)}{\le }\sum _{(s, s') \in {\mathcal {R}}} \mu (s) = \sum _{s' \in B}\sum _{s \in \mathcal R^{-1}(s')}\mu (s)\overset{(ii)}{\le }\sum _{s' \in B}k\mu (s') = k\mu (B)\,. \end{aligned}$$

Before we state the lemmata that are needed to invoke Lemma 5.1, let us discuss the motivation behind them. Let us take the example of the proof of Lemma 4.3 (Lemma 4.4 is very similar). Our goal is to apply the previous lemma for *A* being the event under consideration in the lemma, and *B* the full space of pairs of currents. Since a relation can be viewed as a multi-valued map, we can specify a relation on pairs of currents by describing several ways of modifying a given pair of currents $(\mathbf n_1, \mathbf n_2)$. In order to do so, we first perform the following two steps:

- Select a family *Z* of blocks $\mathbf{B}\in \mathcal B_n(\Lambda _{N})$ that all intersect both ${\mathcal {C}}_{\mathbf n_1}(y)$ and ${\mathcal {C}}_{\mathbf n_1 + \mathbf n_2}(x)$. The number of choices for *Z* will guarantee that *K* is large.
- For each block $\mathbf{B}$, change the value of $\mathbf n_2$ along the edges of a shortest path $\Pi _\mathbf{B}$ between ${\mathcal {C}}_{\mathbf n_1}(y) \cap \mathbf{B}$ and ${\mathcal {C}}_{\mathbf n_1 + \mathbf n_2}(x) \cap \mathbf{B}$ so that these two sets become connected in $\mathbf{B}$ by the resulting pair of currents.

Let us call the new pair of currents $(\mathbf n_1', \mathbf n_2')$ (note that $\mathbf n_1' = \mathbf n_1$ at this stage). We want to be able to recover *Z* from $(\mathbf n_1', \mathbf n_2')$ to ensure a small value of *k* in Property (ii) of Lemma 5.1 (called the *reconstruction step* below). Since *x* and *y* are not connected in $\Lambda _N$ by $\mathbf n_1 + \mathbf n_2$, a natural guess for *Z* would include any block $\mathbf{B}$ containing a vertex $v \in {\mathcal {C}}_{\mathbf n_1}(y)$ which is connected to *x* by $\mathbf {n}'_1+\mathbf {n}'_2$ through a path in $(\Lambda _N{\setminus } {\mathcal {C}}_{\mathbf n_1'}(y)) \cup \{v\}$. Unfortunately, this guess may not be correct because changing $\mathbf n_2$ could have created many such vertices *v* apart from the endpoints of the paths $\Pi _\mathbf{B}$.

One way to address this problem is to additionally (and brutally) require that $(\mathbf n_1' + \mathbf n_2')(e) = 0$ for all the edges *e* adjacent to the paths $\Pi _\mathbf{B}$ except those adjacent to their endpoints, but this has the disadvantage of introducing new sources to the currents. In order to remove these sources, we change the value of $(\mathbf n_1', \mathbf n_2')$ along some new paths connecting pairs of sources. These new paths should avoid the paths $\Pi _\mathbf{B}$ except, possibly, their endpoints in ${\mathcal {C}}_{\mathbf n_1}(y)$ and ${\mathcal {C}}_{\mathbf n_1 + \mathbf n_2}(x)$ so that we do not create any additional connections between ${\mathcal {C}}_{\mathbf n_1}(y)$ and *x*. Furthermore, we want to find these paths in neighborhoods of fixed radius around the paths $\Pi _\mathbf{B}$ to prevent that the value of $(\mathbf n_1, \mathbf n_2)$ is changed on too many edges as that would, again, give a large value of *k* in the reconstruction step.

**Fig. 2:**
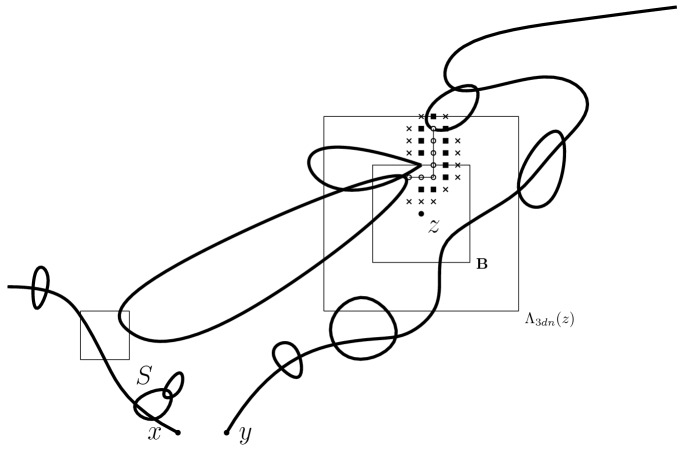
**A schematic version of Lemma** 5.2. The thick lines represent the edges in the multigraph underlying $\mathbf n_1 + \mathbf n_2$ whereas the thin line represents the path $\Pi _\mathbf{B}=(v_0,\dots ,v_k)$. We also depicted the sets $T_\mathbf{B}$ (filled squares) and $S_\mathbf{B} {\setminus } T_\mathbf{B} \cup \{v_k\}$ (crosses)

Our next lemma shows that it is always possible to find such paths within a graph distance of at most 2 from the paths $\Pi _\mathbf{B}$ for some specific choices of the latter. Due to purely technical reasons which we will leverage in our proof of Lemma 4.3, we prove this lemma for ${\mathcal {C}}_{\mathbf n_1 + \mathbf n_2}(S)$ (with *S* an arbitrary set) instead of $\mathcal C_{\mathbf n_1 + \mathbf n_2}(x)$. Without loss of generality, we implicitly assume in the rest of this paper that the size parameters *N* and *n* are integer powers of 2. Also we will use *distance* for the $\ell _\infty$ distance and *graph distance* for the $\ell _1$ distance on $\mathbb {Z}^d$. We say that *A* is connected in *B* if any two vertices of *A* can be connected by a path of vertices in *B*. We refer the reader to Fig. 2 for a pictorial description of Lemma 5.2.

### Lemma 5.2

Let $S \subset \Lambda _N$ be such that ${\mathcal {C}}_{\mathbf n_1}(y) \cap {\mathcal {C}}_{\mathbf n_1 + \mathbf n_2}(S) = \emptyset$. Assume that there exists a block $\mathbf{B}\in {\mathcal {B}}_n(\Lambda _N)$ intersecting ${\mathcal {C}}_{\mathbf n_1 + \mathbf n_2}(S)$ and containing a vertex in ${\mathcal {C}}_{\mathbf n_1}(y)$ with at least two neighbors in $\mathcal C_{\mathbf n_1}(y)$. Then, there exists a path $\Pi _\mathbf{B}=\Pi _\mathbf{B} (\mathbf {n}_1,\mathbf {n}_2)=(v_0, v_1, \ldots , v_{k})$ satisfying that

- $v_0\in {\mathcal {C}}_{\mathbf n_1 + \mathbf n_2}(S)$ is within a distance of at most 3*dn* of the center of $\mathbf{B}$,
- $v_{k} \in {\mathcal {C}}_{\mathbf n_1}(y)$ is within a distance of at most 3*dn* of the center of $\mathbf{B}$,
- $\Pi _\mathbf{B}$ is a shortest path between $v_0$ and $v_k$,
- For every $$0< i <k$$, $$v_i \notin {\mathcal {C}}_{\mathbf n_1}(y) \cup {\mathcal {C}}_{\mathbf n_1 + \mathbf n_2}(S)$$,
- The set $T_\mathbf{B} = T_\mathbf{B}(\mathbf n_1, \mathbf n_2)$ of vertices in $\Lambda _N{\setminus } {\mathcal {C}}_{\mathbf n_1 + \mathbf n_2}(S)$ at a graph distance exactly 1 of $\Pi _\mathbf{B}{\setminus }\{v_0\}$ is connected in the set $S_\mathbf{B}=S_\mathbf{B}(\mathbf n_1, \mathbf n_2)$ of vertices of $\Lambda _N{\setminus } {\mathcal {C}}_{\mathbf n_1 + \mathbf n_2}(S)$ which are either equal to $v_k$ or at a graph distance 1 or 2 of $\Pi _\mathbf{B}$.

We will prove Lemma 5.2 (which is fairly technical but quite clear conceptually) at the end of the paper. With this lemma at our disposal, we can now formally describe the modification of $(\mathbf n_1, \mathbf n_2)$ that we discussed earlier. This is the content of the following lemma.

### Remark 5.3

The requirement that the block $\mathbf{B}$ contains a vertex of ${\mathcal {C}}_{\mathbf {n}_1}(y)$ having two neighbors in ${\mathcal {C}}_{\mathbf {n}_1}(y)$ is used in this lemma and in this lemma only.

We introduce a few notation. From now on, we say that two blocks $\mathbf{B}$ and $\mathbf{B'}$ in ${\mathcal {B}}_n(\Lambda _N)$ are strongly disjoint if their centers are at a distance of 7*dn* of each other. Also, let $N_r(S)$ be the set of vertices of $\mathbb {Z}^d$ within a graph distance at most *r* of *S*, and $E_r(S)$ be the set of edges with both endpoints in $N_r(S)$.

### Lemma 5.4

Assume that $\partial \mathbf n_1 = \{x, y\}$ and $\partial \mathbf n_2 = \emptyset$. Let $S \subset \Lambda _N$ be such that ${\mathcal {C}}_{\mathbf n_1 + \mathbf n_2} (x) \cap S \ne \emptyset$ and ${{\mathcal {C}}_{\mathbf n_1 + \mathbf n_2} (y)} \cap S = \emptyset$. Let *Z* be a family of strongly disjoint blocks of ${\mathcal {B}}_n(\Lambda _{N})$ with the property that every $\mathbf{B}\in Z$ intersects ${\mathcal {C}}_{\mathbf n_1 + \mathbf n_2}(S)$ and contains a vertex of ${\mathcal {C}}_{\mathbf n_1}(y)$ having at least two neighbors in ${\mathcal {C}}_{\mathbf n_1}(y)$.

Then, there exists a new pair of currents $(\mathbf n_{1}', \mathbf n_{2}')$ (which is a function of $\mathbf {n}_1$, $\mathbf {n}_2$ and *Z*) with the following properties. If the paths $\Pi _\mathbf{B} = \Pi _\mathbf{B}(\mathbf n_1, \mathbf n_2) = (v_0^\mathbf{B}, v_1^\mathbf{B}, \ldots , v_{k_\mathbf{B}}^\mathbf{B})$ are given by Lemma 5.2 above, we have that

- $$\partial \mathbf n_1' = \partial \mathbf n_1$$ and $$\partial \mathbf n_2' = \partial \mathbf n_2$$.
- $(\mathbf n_1, \mathbf n_2)(e)\ne (\mathbf n_1', \mathbf n_2')(e)$ implies that $e\in E_2(\Pi _\mathbf{B})$ for some $\mathbf{B}\in Z$.
- $(\mathbf n_1, \mathbf n_2)(e)\ne (\mathbf n_1', \mathbf n_2')(e)$ implies that $\mathbf n_j'(e) \le \mathbf n_j(e)$ or $\mathbf n_j'(e) \le 2$ for $j=1,2$.
- The set of vertices $v \in {\mathcal {C}}_{\mathbf n_{1}'}(y)$ that are connected by $\mathbf {n}'_1+\mathbf {n}'_2$ to *S* in $(\Lambda _N {\setminus } {\mathcal {C}}_{\mathbf n_{1}'(y)}) \cup \{v\}$ is exactly equal to the set of endpoints $v_{k_\mathbf{B}}^\mathbf{B}$ of the paths $\Pi _\mathbf{B}$ with $\mathbf{B}\in Z$.

We already explained why Properties (b) and (d) are important for the reconstruction step. Property (c) is motivated by the observation that

$$\begin{aligned} \sum _{\mathbf n: \mathbf n \ge \mathbf n', \mathbf n_{|{\mathsf {E}}} = \mathbf n'_{|{\mathsf {E}}}}\frac{w(\mathbf n)}{w(\mathbf n')} \le \mathrm {e}^{C|{\mathsf {E}}|} \end{aligned}$$

for some $C > 0$ and any ${\mathsf {E}} \subset E_N$, and thus is crucial for efficient reconstruction. We will prove Lemma 5.4 slightly later, but before that let us show how can it can be used to derive Lemmata 4.3 and 4.4.

### Proof of Lemma 4.3

The main idea underlying the proof is the following. We already know from Proposition 4.1 that $\mathbf{B}$ and $\mathbf{B}'$ are connected in $\mathbf n_1 + \mathbf n_2$ for every $\mathbf{B},\mathbf{B'}\in {\mathcal {B}}_n(\Lambda _N)$ with an extremely high probability. As a consequence, we can effectively assume that this happens. Then, it is easy to see that when ${\mathcal {C}}$ occurs and *x*, *y* are not connected in $\Lambda _N$ by $\mathbf n_1 + \mathbf n_2$, one can find a set *S* and at least *N* / (4*n*) many blocks $\mathbf{B} \in {\mathcal {B}}_n(\Lambda _N)$ satisfying the conditions of Lemma 5.2 (which are the same as the conditions on the elements of *Z* in Lemma 5.4). Thus, we can pick any subset *Z* of a certain number of such blocks with the additional condition that they are strongly disjoint, and modify $(\mathbf n_1, \mathbf n_2)$ according to the previous lemma. This gives us a relation on the set of pairs of currents and allows us to apply Lemma 5.1 for bounding the probability of the event we are interested in. As we will see later in the proof, the bound we obtain involves the (fixed) size of *Z* as a parameter and the lemma follows by choosing an appropriate value for this size. The detailed argument is given below.

**Construction of**${\mathcal {R}}$. Let $V(\mathbf n_1, \mathbf n_2)$ be a maximal subset of strongly disjoint blocks $\mathbf{B}\in {\mathcal {B}}_n(\Lambda _N)$ with the property that ${\mathcal {C}}_{\mathbf n_1 + \mathbf n_2}(x) \cap \mathbf{B} = \emptyset$ and $\mathbf{B}$ contains a vertex of ${\mathcal {C}}_{\mathbf n_1}(y)$ having at least two neighbors in ${\mathcal {C}}_{\mathbf n_1}(y)$. Let ${\mathcal {C}}_m$ denote the sub-event of ${\mathcal {C}}$ for which $V(\mathbf n_1, \mathbf n_2)$ contains exactly *m* blocks. Also, let ${\mathcal {E}}$ be the event that every two blocks $\mathbf{B}$ and $\mathbf{B}'$ in ${\mathcal {B}}_n(\Lambda _N)$ are connected by $\mathbf {n}_1+\mathbf {n}_2$ in $\Lambda _N$. Finally, define

**Fig. 3:**
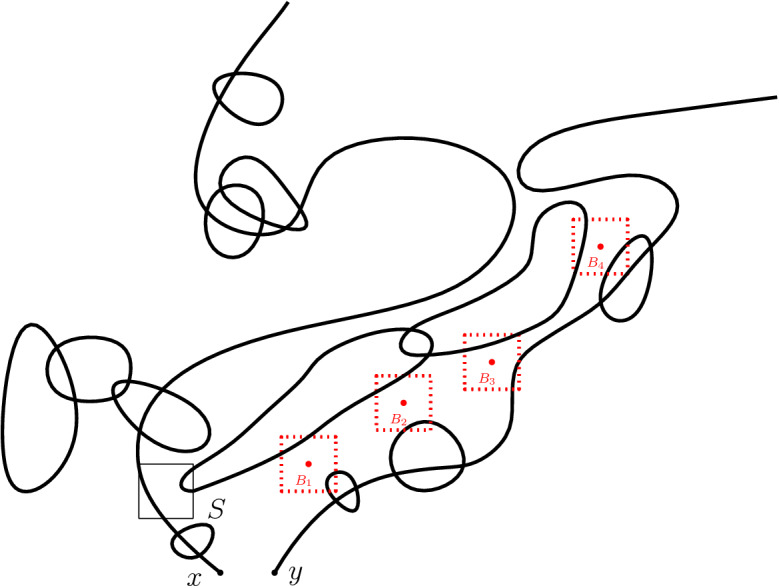
A simple representation of the set $V(\mathbf n_1, \mathbf n_2)$

Let $S = S(\mathbf n_1, \mathbf n_2) \in {\mathcal {B}}_n(\Lambda _N)$ be a block intersecting ${\mathcal {C}}_{\mathbf n_1}(x)$ but not ${\mathcal {C}}_{\mathbf n_1 + \mathbf n_2}(y)$. We remark that such a set *S* exists since ${\mathcal {D}}_m \subset {\mathcal {C}}\subset {\mathcal {C}}_{y,x}$.

The conditions of Lemma 5.4 are met for *S* and any subset *Z* of $V(\mathbf n_1, \mathbf n_2)$. Therefore, we can define a relation as follows. Fix $\delta >0$ to be a small number to be determined later. Then, $((\mathbf n_1, \mathbf n_2),(\mathbf n_1', \mathbf n_2'))\in {\mathcal {R}}$ if and only if $(\mathbf {n}_1,\mathbf {n}_2)\in {\mathcal {D}}_m$ and

$$\begin{aligned} (\mathbf {n}_1' ,\mathbf {n}'_2)= (\mathbf n_{1}',\mathbf {n}'_2)(\mathbf n_1, \mathbf n_2, Z)\text { for some }Z \subset V(\mathbf n_1, \mathbf n_2)\text { such that }|Z| = \delta m, \end{aligned}$$

where the map $(\mathbf {n}_1,\mathbf {n}_2,Z)\mapsto (\mathbf {n}'_1,\mathbf {n}_2')$ is given by Lemma 5.4.

With this definition of ${\mathcal {R}}$, we can now check Properties (i) and (ii) of Lemma 5.1 to deduce a bound on the probability of ${\mathcal {D}}_m$.

**Property (i).** For any $(\mathbf n_1, \mathbf n_2)\in {\mathcal {D}}_m$, the map $Z \mapsto \{v_{k_\mathbf{B}}^\mathbf{B}: \mathbf{B} \in Z\}$ is one-to-one since $v^\mathbf{B}_{k_\mathbf{B}}$ is within a distance 3*dn* of the center of $\mathbf{B}$, and the blocks in $V(\mathbf n_1, \mathbf n_2)$ are strongly disjoint. Hence,

5.1 $$\begin{aligned} |{\mathcal {R}}((\mathbf n_1, \mathbf n_2))| \ge \left( {\begin{array}{c}|V(\mathbf n_1, \mathbf n_2)|\\ \delta m\end{array}}\right) =\left( {\begin{array}{c}m\\ \delta m\end{array}}\right) \,. \end{aligned}$$

**Property (ii).** Fix $(\mathbf m_1, \mathbf m_2)$. For a block *S*, a set of $\delta m$ strongly disjoint blocks *Z*, a subset of edges *E* and a collection of paths $\Pi =(\Pi _\mathbf{B}:\mathbf{B}\in Z)$, introduce the set ${\mathcal {A}}(\mathbf m_1,\mathbf m_2,S,Z,E,\Pi )$ of pairs of currents $(\mathbf {n}_1,\mathbf {n}_2)\in {\mathcal {D}}_m$ such that

- $$S(\mathbf {n}_1,\mathbf {n}_2)=S$$,
- $$(\mathbf m_1,\mathbf m_2) =(\mathbf n_1',\mathbf {n}'_2) (\mathbf {n}_1,\mathbf {n}_2,Z)$$,
- $$E=\{e\in E_N:(\mathbf {n}_1,\mathbf {n}_2)(e)\ne (\mathbf m_1,\mathbf m_2)(e)\}$$,
- $$\Pi _\mathbf{B}(\mathbf {n}_1,\mathbf {n}_2)=\Pi _\mathbf{B}$$ for every $$\mathbf{B}\in Z$$.

The definition of ${\mathcal {R}}$ implies directly that

5.2 $$\begin{aligned} \sum _{(\mathbf n_1, \mathbf n_2) \in {\mathcal {R}}^{-1}(\mathbf m_1, \mathbf m_2)}&{\mathbb {P}}_{\Lambda _N^+, \Lambda _N}^{\{x, y\}, \emptyset }[(\mathbf n_1, \mathbf n_2)] \nonumber \\&= {\mathbb {P}}_{\Lambda _N^+, \Lambda _N}^{\{x, y\}, \emptyset }[(\mathbf m_1, \mathbf m_2)]\sum _{(S, Z, E, \Pi )}\sum _{(\mathbf n_1, \mathbf n_2) \in {\mathcal {A}}(\mathbf m_1,\mathbf m_2,S,Z,E,\Pi )} \frac{w(\mathbf n_1)w(\mathbf n_2)}{w(\mathbf m_1)w(\mathbf m_2)}. \end{aligned}$$

We will bound the two summations on the right of (5.2) in two steps. First, Properties (b) and (c) of Lemma 5.4 imply that

5.3 $$\begin{aligned}&\sum _{(\mathbf n_1, \mathbf n_2) \in {\mathcal {A}}(\mathbf m_1,\mathbf m_2,S,Z,E,\Pi )} \frac{w(\mathbf n_1)w(\mathbf n_2)}{w(\mathbf m_1)w(\mathbf m_2)}\nonumber \\&\qquad \le \prod _{\begin{array}{c} e \in E\\ j=1,2 \end{array}}\Big (\sum _{\ell \ge \mathbf m_j(e)}\frac{\beta ^{\ell }\mathbf m_j(e)!}{\beta ^{\mathbf m_j(e)}\ell !} + \sum _{0 \le \ell \le \mathbf m_j(e)}\frac{\beta ^{\ell }\mathbf m_j(e)!}{\beta ^{\mathbf m_j(e)}\ell !}{\mathbb {I}}[\mathbf m_j(e) \le 2]\Big )\nonumber \\&\qquad \le \exp ({C|E|})\le \exp (C\delta mn). \end{aligned}$$

In the second line, we used that *E* is included in the union of the $E_2(\Pi _\mathbf{B})$ for $\mathbf{B}\in Z$. Furthermore, $E_2(\Pi _\mathbf{B})$ is the set of vertices at a graph distance at most 2 from $\Pi _\mathbf{B}$. According to Lemma 5.2, $\Pi _\mathbf{B}$ must be a shortest length path between two vertices within a distance 3*dn* of the center of $\mathbf{B}$, a fact which implies that its length is smaller than $6d^2n$. Overall, this implies that $|E|\le 6d^2n|Z|\le C\delta mn$.

Second, we bound the number of possibilities for *S*, *Z*, *E* and $\Pi$. Obviously, there are fewer than $|{\mathcal {B}}_n(\Lambda _N)|$ choices for *S*. Property (d) of Lemma 5.4 implies that $(\mathbf m_1,\mathbf m_2)$ and *S* determine the points $v_{k_\mathbf{B}}^\mathbf{B}$ for $\mathbf{B}\in Z$. Since these vertices are within a distance 3*dn* of the centers of the blocks in *Z*, and that there are $\delta m$ blocks in *Z*, this reduces the number of possibilities for *Z* to $(2d)^{3d\delta m}$. Also, each one of the paths $\Pi _\mathbf{B}$ is a self-avoiding path of length at most $6d^2n$ ending at $v_{k_\mathbf{B}}^\mathbf{B}$, and therefore there are at most $(2d)^{6d^2n\delta m}$ choices for the collection of paths $\Pi _\mathbf{B}$ with $\mathbf{B}\in Z$. Finally, *E* being a subset of $\cup _{\mathbf{B} \in Z}E_2(\Pi _\mathbf{B})$, we deduce that the number of possibilities for *E* is bounded by $2^{25d^3n\delta m}$.

Overall plugging these bounds and (5.3) into (5.2) gives

$$\begin{aligned} \sum _{(\mathbf n_1, \mathbf n_2) \in {\mathcal {R}}^{-1}(\mathbf m_1, \mathbf m_2)}{\mathbb {P}}_{\Lambda _N^+, \Lambda _N}^{\{x, y\}, \emptyset }[(\mathbf n_1, \mathbf n_2)] \le N^d\mathrm {e}^{C\delta mn}\,{\mathbb {P}}_{\Lambda _N^+, \Lambda _N}^{\{x, y\}, \emptyset }[(\mathbf m_1, \mathbf m_2)]\,. \end{aligned}$$

**Conclusion of the proof.** Plugging the last inequality and (5.1) in Lemma 5.1 gives

5.4 $$\begin{aligned} {\mathbb {P}}^{\{x,y\}, \emptyset }_{\Lambda _N^+, \Lambda _N}[{\mathcal {D}}_m] \le \frac{CN^d\mathrm {e}^{C\delta mn}}{\left( {\begin{array}{c}m\\ \delta m\end{array}}\right) }\,. \end{aligned}$$

At this stage, recall that $N=e^{n^\alpha }$ with $\alpha >1$. An elementary computation shows that choosing $\delta = \mathrm {e}^{-2Cn}$ (which is a valid choice since $\delta m \ge \mathrm {e}^{-2Cn} N / (4n) = \mathrm {e}^{n^\alpha - 2Cn} / (4n) > 1$ for every large *n*) gives that

$$\begin{aligned} {\mathbb {P}}^{\{x, y\}, \emptyset }_{\Lambda _N^+, \Lambda _N}[{\mathcal {D}}_m] \le \mathrm {e}^{-c\mathrm {e}^{-Cn} mn}\, \end{aligned}$$

for every $m \ge \tfrac{1}{4(2d)^{4d}}N/n$ and *N* large enough (independent of *m*). Since any pair $(\mathbf {n}_1,\mathbf {n}_2)\in {\mathcal {C}}$ must be in one of the ${\mathcal {C}}_m$ for $m \ge \tfrac{1}{4(2d)^{4d}}N/n$, we obtain that5.5by summing over *m*. Now, since $N = \mathrm {e}^{n^\alpha }$ for $\alpha < d-1$, it follows from Proposition 4.1 that

5.6 $$\begin{aligned} {\mathbb {P}}_{\Lambda _N^+, \Lambda _N}^{\{x, y\}, \emptyset }[{\mathcal {E}}] \ge 1 - CN^d\mathrm {e}^{-cn^{d-1}} \ge 1 - \mathrm {e}^{-cn^{d-1}}\, \end{aligned}$$

for every large enough *N*. The result follows readily from this bound and (5.5). $\quad \square$

### Remark 5.5

We already used the bound $\alpha <d-1$ to deduce Proposition 1.5 from Lemmata 4.3 and 4.4. The end of the previous proof further explains where $1<\alpha <d-1$ is used. Indeed, $\alpha <d-1$ is used to invoke Proposition 4.1. The bound $\alpha >1$ is there to guarantee that the exponential (in *n*) finite-energy cost appearing for instance in (5.3) is overcome by the choice of *N*.

### Proof of Lemma 4.4

The proof is the same as the previous one, with $S=\{x\}$ instead of *S* being a block. $\quad \square$

We now focus on the proof of Lemma 5.4 using Lemma 5.2.

### Proof of Lemma 5.4

We begin with the construction of $(\mathbf {n}_1',\mathbf {n}'_2)$ and then show that Properties (a)–(d) are satisfied.

**Construction of **$(\mathbf {n}_{1}', \mathbf {n}_{2}')$. We proceed in three steps roughly corresponding to the steps that we outlined before Lemma 5.2, albeit in a slightly different order. For clarity of exposition, we will use different notations for the pairs of currents resulting from different steps. As a result, we construct three intermediate currents called $\mathbf {n}_1^0$, $\mathbf {n}_2^0$, and $\mathbf {n}_2^1$. At the conclusion of each step, we track the sources of the currents constructed in that step as well as the direction in which their value changes from the previous step. This will help us to verify Properties (a) and (c) at the end.

Also, since the construction will obviously be independent for every $\mathbf{B}\in Z$ (since the blocks are strongly disjoint), we present it only near one prescribed block $\mathbf{B}$, which we remove from the notation for convenience. We therefore write $\Pi =(v_0,\dots ,v_k)$. Also, we set $\overline{\Pi }=(v_1,\dots ,v_{k-1})$ to be the path $\Pi$ minus its endpoints. We will return to the original notation later when we verify the properties of $(\mathbf n_1', \mathbf n_2')$.

*Step 1: Closing the edges adjacent to *$\Pi$. We want to make both currents 0 on edges adjacent to $\Pi$ except those adjacent to its endpoints. Formally, set

5.7 $$\begin{aligned} \mathbf n_{1}^0 (e)&:= {\left\{ \begin{array}{ll} 0 &{}\quad \text{ if } e = \{v, w\} \text{ for } v\in {\overline{\Pi }}\hbox { and } w\in \Lambda _N, \\ \mathbf n_1(e) &{}\quad \text {otherwise}, \end{array}\right. } \end{aligned}$$

5.8 $$\begin{aligned} \mathbf n_{2}^0 (e)&:= {\left\{ \begin{array}{ll} 0 &{}\quad \text{ if } e = \{v,w\} \text{ for } v\in {\overline{\Pi }}\hbox { and } w\in \Lambda _N{\setminus } \Pi , \\ \mathbf n_2(e) &{}\quad \text {otherwise}\,. \end{array}\right. } \end{aligned}$$

In order to track the sources of $\mathbf n_1^0$, let us first notice that vertices in ${\overline{\Pi }}$ are not sources since they are incident to edges with zero $\mathbf {n}_1^0$ current only. Since ${\overline{\Pi }}$ does not intersect ${\mathcal {C}}_{\mathbf n_1}(y)$ or ${\mathcal {C}}_{\mathbf n_1 + \mathbf n_2}(S)$ (by the definition of $\Pi$ in Lemma 5.2), we deduce that the sources of $\mathbf {n}_1^0$ not equal to *x* or *y* and within a distance of 4*dn* of the center of $\mathbf{B}$ must be at a distance exactly equal to 1 of ${\overline{\Pi }}$ and that at least one of the edges incident to them must have changed value between $\mathbf {n}_1$ and $\mathbf {n}_1^0$. Since edges between ${\overline{\Pi }}$ and ${\mathcal {C}}_{\mathbf n_1 + \mathbf n_2}(S) \cup {\mathcal {C}}_{\mathbf n_1}(y)$ already had a zero current in $\mathbf {n}_1$, we deduce that the new sources cannot be in ${\mathcal {C}}_{\mathbf {n}_1+\mathbf {n}_2}(S)$ and must therefore be in the set $T_\mathbf{B} = T_\mathbf{B}(\mathbf n_1, \mathbf n_2)$ defined in Lemma 5.2. Also, notice that there is an even number of such sources. Similarly, we get that the set of sources of $\mathbf {n}_2^0$ is a subset of ${\overline{\Pi }}\cup T_\mathbf{B}$.

Finally, notice that

5.9 $$\begin{aligned} \mathbf n_{1}^0(e) \le \mathbf n_1(e) \text{ and } \mathbf n_{2}^0(e) \le \mathbf n_2(e) \text{ for } \text{ every } e \in E_N\,. \end{aligned}$$

*Step 2. Opening the edges along *$\Pi$. The second step consists in defining $\mathbf {n}_1^1=\mathbf {n}_1^0$ and

$$\begin{aligned} \mathbf n_{2}^1 (e): = {\left\{ \begin{array}{ll} 2 &{}\quad \text{ if } e = \{v, w\} \text{ with } v,w\in \Pi \text{ or } \text{ if } v = v_k \text{ and } w \in \Lambda _N.\\ \mathbf n_{2}^0(e) &{}\quad \text {otherwise}. \end{array}\right. } \end{aligned}$$

By definition,

5.10 $$\begin{aligned} \mathbf n_{2}^1(e) \ne \mathbf n_{2}^0(e) \hbox { implies that }\mathbf n_{2}^1(e) = 2. \end{aligned}$$

Since ${\overline{\Pi }}$ does not intersect ${\mathcal {C}}_{\mathbf n_1 + \mathbf n_2}(S)$, we have $\mathbf n_2(\{v_{k-1}, v_k\}) = \mathbf n_2^0(\{v_{k-1}, v_k\}) = 0$ and therefore the definition of $\mathbf n_2^1$ also implies that a source of $\mathbf n_2^1$ is a source of $\mathbf {n}_2^0$ which is not on $\Pi$. Hence, it is included in $T_\mathbf{B}$ again and also, there are an even number of sources within a distance of 4*dn* of $\mathbf{B}$.

*Step 3. Killing the sources of *$(\mathbf n_1^1, \mathbf n_2^1)$. We now remove the additional sources of $\mathbf n_1^1$ and $\mathbf n_2^1$, all of which lie in $T_\mathbf{B}$. We start with the sources of $\mathbf {n}_1^1$. By the fifth item of Lemma 5.2, $T_\mathbf{B}$ is a connected subset of $S_\mathbf{B}$. We can therefore choose (in some arbitrary but fixed manner) paths $\Gamma _{1}^1, \ldots , \Gamma _{\ell }^1$ in $S_\mathbf{B}$ pairing the sources of $\mathbf {n}_1^1$. Let $\mathbf m$ be the current, equal at each edge to 0 (resp. 1) if there is an even (resp. odd) number of paths going through this edge. Finally, set

$$\begin{aligned} \mathbf n_1'(e) := {\left\{ \begin{array}{ll} \mathbf n_1^1(e) - \mathbf m(e) &{}\quad \text{ if } \mathbf n_1^1(e) \ge 2\\ \mathbf n_1^1(e) + \mathbf m(e) &{}\quad \text {otherwise}\,. \end{array}\right. } \end{aligned}$$

We obtain immediately that $\partial \mathbf n_1'=\{x,y\}$. We proceed in the same way for $\mathbf {n}_2^1$ in order to obtain $\mathbf {n}_2'$ with $\partial \mathbf {n}_2'=\emptyset$. Again, notice that

5.11 $$\begin{aligned} \mathbf n_{j}'(e) \le \mathbf n_j^1(e) \text{ or } \mathbf n_{j}'(e) \le 2 \text{ for } \text{ every } e \in E_N \text{ and } j = 1,2\,. \end{aligned}$$

**Verification of the properties of **$(\mathbf n_1', \mathbf n_2')$. In this part, we assume that we made the construction above for every $\mathbf{B}\in Z$.

*Property (a)* This follows readily from the two sentences preceding (5.11).

*Property (b)* At each stage of the construction, edges for which the value of currents is modified are always within a graph distance 2 from one of the paths $\Pi _\mathbf{B}$, which means that Property (b) is satisfied.

*Property (c)* This follows from the displays (5.9), (5.10) and (5.11).

*Property (d)* Let us first verify one direction of Property (d), namely that for any $\mathbf B \in Z$, $v_{k_\mathbf{B}}^\mathbf{B} \in {\mathcal {C}}_{\mathbf n_1'}(y)$ and it is connected in $(\Lambda _N {\setminus } {\mathcal {C}}_{\mathbf n_{1}'(y)}) \cup \{v_{k_\mathbf{B}}^\mathbf{B}\}$ to *S* by $\mathbf n_1' + \mathbf n_2'$. We divide the proof into two steps.

*Proof that *$v_{k_\mathbf{B}}^\mathbf{B} \in {\mathcal {C}}_{\mathbf n_1'}(y)$. It suffices to show that ${\mathcal {C}}_{\mathbf n_1}(y) \subset {\mathcal {C}}_{\mathbf n_1'}(y)$. It is clear from our construction in Step 3 that ${\mathcal {C}}_{\mathbf n_1^0}(y) = {\mathcal {C}}_{\mathbf n_1^1}(y) \subset {\mathcal {C}}_{\mathbf n_1'}(y)$. Also, the definition of $\mathbf n_1^0$ implies that $\mathbf n_1^0(e) < \mathbf n_1(e)$ only if $e = \{v, w\}$ for $v \in {\overline{\Pi }}_{\mathbf{B}}$ where $\mathbf B \in Z$ and $w \in \Lambda _N$. Since ${\overline{\Pi }}_\mathbf{B}$ does not intersect ${\mathcal {C}}_{\mathbf n_1}(y)$ by Lemma 5.2, we therefore get ${\mathcal {C}}_{\mathbf n_1^0}(y) = {\mathcal {C}}_{\mathbf n_1}(y)$ which completes the proof.

*Proof that *$v_{k_\mathbf{B}}^\mathbf{B}$*is connected in *$(\Lambda _N {\setminus } {\mathcal {C}}_{\mathbf n_{1}'(y)}) \cup \{v_{k_\mathbf{B}}^\mathbf{B}\}$*to Sby *$\mathbf n_1' + \mathbf n_2'$. Notice that the path $\Pi _\mathbf{B}$ is open in $\mathbf n_2'$ and connects $v_{k_\mathbf{B}}^\mathbf{B}$ to $v_0^\mathbf{B}$. Since $v_0^\mathbf{B} \in {\mathcal {C}}_{\mathbf n_1 + \mathbf n_2}(S)$ by definition, it therefore suffices to show that ${\mathcal {C}}_{\mathbf n_1 + \mathbf n_2}(S) \subset {\mathcal {C}}_{\mathbf n_1' + \mathbf n_2'}(S)$ and that ${\overline{\Pi }}_\mathbf{B}$ and ${\mathcal {C}}_{\mathbf n_1 + \mathbf n_2}(S)$ do not intersect ${\mathcal {C}}_{\mathbf n_1'}(y)$.

To this end, let us first recall from the definitions of $(\mathbf n_{1}^1, \mathbf n_{2}^1)$ and $(\mathbf n_{1}', \mathbf n_{2}')$ that $(\mathbf n_1' + \mathbf n_2')(e) > 0$ whenever $(\mathbf n_1^0 + \mathbf n_2^0)(e) > 0$, and consequently ${\mathcal {C}}_{\mathbf n_{1}^0 + \mathbf n_{2}^0}(S)\subset {\mathcal {C}}_{\mathbf n_{1}' + \mathbf n_{2}'}(S)$. Also, from the construction in Step 1 we have $(\mathbf n_1^0 + \mathbf n_2^0)(e) < (\mathbf n_1 + \mathbf n_2)(e)$ only if *e* has an endpoint in ${\overline{\Pi }}_{\mathbf B}$ for some $\mathbf B \in Z$. Since ${\overline{\Pi }}_\mathbf{B}$ does not intersect ${\mathcal {C}}_{\mathbf n_1 + \mathbf n_2}(S)$ by Lemma 5.2, it therefore follows that ${\mathcal {C}}_{\mathbf n_{1}^0 + \mathbf n_{2}^0}(S) = {\mathcal {C}}_{\mathbf n_{1} + \mathbf n_{2}}(S)$ and hence ${\mathcal {C}}_{\mathbf n_1 + \mathbf n_2}(S) \subset {\mathcal {C}}_{\mathbf n_1' + \mathbf n_2'}(S)$.

Next let us “bound” the set ${\mathcal {C}}_{\mathbf n_1'}(y)$ from above. Notice that

$$\begin{aligned} {\mathcal {C}}_{\mathbf n_{1}'}(y)&\subset {\mathcal {C}}_{\mathbf n_{1}^0}(y) \cup \bigcup _{\mathbf{B} \in Z}{\mathcal {C}}_{\mathbf n_{1}^0}(S_\mathbf{B}) = {\mathcal {C}}_{\mathbf n_1}(y) \cup \bigcup _{\mathbf{B} \in Z}{\mathcal {C}}_{\mathbf n_{1}^0}(S_\mathbf{B})\,, \end{aligned}$$

where in the second step we used ${\mathcal {C}}_{\mathbf n_1}(y) = {\mathcal {C}}_{\mathbf n_1^0}(y)$ as proved in the previous part. Since, by Lemma 5.2, ${\overline{\Pi }}_{\mathbf B}$ is disjoint from ${\mathcal {C}}_{\mathbf n_1}(y)$ and $S_{\mathbf B}$, we deduce from the previous displayed equation that ${\mathcal {C}}_{\mathbf n_{1}'}(y) \cap {\overline{\Pi }}_{\mathbf B} = \emptyset$ for any $\mathbf B \in Z$ if we show ${\mathcal {C}}_{\mathbf n_1^0}({\overline{\Pi }}_{\mathbf B})= {\overline{\Pi }}_{\mathbf B}$ for any such $\mathbf B$. But this follows immediately from the fact that $\mathbf n_{1}^0(e) = 0$ for any edge *e* whose one endpoint lies in ${\overline{\Pi }}_\mathbf{B}$ for some $\mathbf{B}\in Z$.

Similarly, from ${\mathcal {C}}_{\mathbf n_1}(y) \cap {\mathcal {C}}_{\mathbf n_1 + \mathbf n_2}(S) = \emptyset$ and $S_{\mathbf B} \cap {\mathcal {C}}_{\mathbf n_1 + \mathbf n_2}(S) = S_{\mathbf B} \cap {\mathcal {C}}_{\mathbf n_1^0 + \mathbf n_2^0}(S) = \emptyset$ (both are consequences of Lemma 5.2), we deduce that ${\mathcal {C}}_{\mathbf n_1'}(y) \cap {\mathcal {C}}_{\mathbf n_1 + \mathbf n_2}(S) = \emptyset$, concluding the proof of this part.

It remains to verify the other direction of Property (d), namely that any vertex $v \in {\mathcal {C}}_{\mathbf n_{1}'}(y)$ that is connected to *S* by $\mathbf {n}'_1+\mathbf {n}'_2$ in $(\Lambda _N {\setminus } {\mathcal {C}}_{\mathbf n_{1}'(y)}) \cup \{v\}$ must be the endpoint $v_{k_\mathbf{B}}^\mathbf{B}$ of a path $\Pi _\mathbf{B}$ for some $\mathbf{B}\in Z$. It suffices to show that any self-avoiding path $\Pi$ in $\mathbf n_{1}' + \mathbf n_{2}'$ between *S* and ${\mathcal {C}}_{\mathbf n_{1}'}(y)$ contains $v_{k_\mathbf{B}}^\mathbf{B}$ for some $\mathbf{B}\in Z$.

Since the blocks are strongly disjoint, we deduce from the definition of $(\mathbf n_{1}', \mathbf n_{2}')$ that

$$\begin{aligned} (\mathbf n_{1}' + \mathbf n_{2}')(e) = (\mathbf n_{1}^1 + \mathbf n_{2}^1)(e) = 0\, \end{aligned}$$

for every edge with one endpoint in some ${\overline{\Pi }}_\mathbf{B}$. Together with the fact that ${\overline{\Pi }}_\mathbf{B}$ is disjoint from ${\mathcal {C}}_{\mathbf n_1'}(y)$ and *S* (already noted in the previous parts), this implies that if $\Pi$ contains an edge of $\Pi _\mathbf{B}$, then it must contain the vertex $v_{k_\mathbf{B}}^\mathbf{B}$. On the other hand, if $\Pi$ does not contain any edge of $\Pi _\mathbf{B}$ for any $\mathbf{B} \in Z$, then it must contain an edge with one endpoint in ${\mathcal {C}}_{\mathbf n_{1} + \mathbf n_{2}}(S)$ and another which is not in ${\mathcal {C}}_{\mathbf n_{1} + \mathbf n_{2}}(S)$ or in any of the ${\overline{\Pi }}_\mathbf{B}$. This is because ${\mathcal {C}}_{\mathbf n_1'}(y) \cap {\mathcal {C}}_{\mathbf n_1 + \mathbf n_2}(S) = \emptyset$ as we noted in the previous part. Obviously such an edge cannot exist in $\mathbf n_1 + \mathbf n_2$. Now, observing that $(\mathbf n_1 + \mathbf n_2)(e) < (\mathbf n_1' + \mathbf n_2')(e)$ only if *e* is an edge in $\Pi _{\mathbf B}$ or $S_{\mathbf B}$ for some $\mathbf B \in Z$ and that $S_{\mathbf B} \cap {\mathcal {C}}_{\mathbf n_1 + \mathbf n_2}(S) = \emptyset$, we conclude that such an edge cannot exist in $\mathbf n_{1}' + \mathbf n_{2}'$ as well, thus finishing the proof.$\quad \square$

Finally, we are left with the proof of Lemma 5.2. It is clear that the only non-trivial part of the lemma is the fifth item. However, the following crucial observation makes it much simpler. Suppose that we choose $\Pi _\mathbf{B}:=(v_0, \ldots , v_k)$ as a shortest path between ${\mathcal {C}}_{\mathbf n_1 + \mathbf n_2}(S)$ and ${\mathcal {C}}_{\mathbf n_1}(y)$ restricted to some region. Also suppose that $N_2(\Pi _{\mathbf B})$ lies in that region. Then the distance between $v_t$ and ${\mathcal {C}}_{\mathbf n_1 + \mathbf n_2}(S)$ is at least 3 for any $t \ge 3$ (since otherwise there would be a shorter path between ${\mathcal {C}}_{\mathbf n_1 + \mathbf n_2}(S)$ and ${\mathcal {C}}_{\mathbf n_1}(y)$ restricted to the region) and hence $N_2(v_t) {\setminus } (\Pi _\mathbf{B} \cup {\mathcal {C}}_{\mathbf n_1 + \mathbf n_2}(S)) = N_2(v_t) {\setminus } \Pi _\mathbf{B}$ for any such *t*. Now, in dimension $d \ge 3$, it is not difficult to prove that $N_1(v_t) {\setminus } \Pi _\mathbf{B}$ is a connected subset of $N_2(v_t) {\setminus } \Pi _\mathbf{B}$ if $\Pi _\mathbf{B}$ is a shortest path between its endpoints. Hence, any two vertices in $N_1(v_t) {\setminus } \Pi _\mathbf{B}$ can be connected using a path in $N_2(v_t) {\setminus } \Pi _\mathbf{B}$ that does not intersect ${\mathcal {C}}_{\mathbf n_1 + \mathbf n_2}(S)$. Therefore the only “problematic” vertices in $T_\mathbf{B}$ (see the statement of Lemma 5.2) are those in $N_1(v_1)$ and $N_1(v_2)$. We deal with them in the proof by carefully considering all possible alignments for the first three edges of $\Pi _\mathbf{B}$.

### Proof of Lemma 5.2

For the purpose of this proof, we use $\mathbf e_j$ to denote the vertex in $\mathbb {Z}^d$ whose *j*-th coordinate is 1 and all the other coordinates are 0. Let *u* be a vertex in ${\mathcal {C}}_{\mathbf n_1}(y) \cap \mathbf{B}$ with at least two neighbors in ${\mathcal {C}}_{\mathbf n_1}(y)$. Since $\mathbf{B}$ intersects ${\mathcal {C}}_{\mathbf n_1 + \mathbf n_2}(S)$, there is a vertex $v \in {\mathcal {C}}_{\mathbf n_1 + \mathbf n_2}(S)$ which is within a distance of 3*dn* from the center of $\mathbf{B}$ realizing the graph distance, denoted by $d_1$ below, between *u* and ${\mathcal {C}}_{\mathbf n_1 + \mathbf n_2}(S)$ restricted to the box of radius 4*dn* with the same center as $\mathbf B$.

Now consider a shortest (for the length) path $\Pi ':=(v = v_0', v_1', \ldots , v_{d_1}' = u)$ between *v* and *u* as our first choice for $\Pi _{\mathbf B}$. For any two vertices *p*, *q* adjacent to $v_t$ which do not lie in $\Pi '$ or ${\mathcal {C}}_{\mathbf n_1 + \mathbf n_2}(S)$ (where $t \ge 1$), we would like to connect them by a path in $\{v'_{d_1}\} \cup N_2(\Pi ') {\setminus } \Pi '$ which avoids ${\mathcal {C}}_{\mathbf n_1 + \mathbf n_2}(S)$. In what follows we do this based on the value of *t*.

When $t \ge 3$, the observation we made before the proof implies that the graph distance between any vertex in $N_2(v_t')$ and *u* is strictly less than $d_1$ and hence $N_2(v_t')$ does not intersect ${\mathcal {C}}_{\mathbf n_1 + \mathbf n_2}(S)$. Thus we only need to show that, for $t \ge 3$, the set $N_1(v_t') {\setminus } \Pi '$ is connected in $N_2(v_t') {\setminus } \Pi '$. To this end we consider two distinct possibilities for a pair of vertices $w, w'$ in $N_1(v_t') {\setminus } \Pi '$. The first possibility is that $w = v_t' + \mathbf e$ and $w' = v_t' + \mathbf e'$ for some $\mathbf e, \mathbf e' \in \{\pm e_j: j \le d\}$ such that $\mathbf e \ne -\mathbf e'$. Notice that in this case, the vertex $v_t' + \mathbf e + \mathbf e'$ cannot lie in $\Pi '$ since otherwise the segment of $\Pi '$ between $v_t'$ and $v_t' + \mathbf e + \mathbf e'$ would contain at least 3 edges contradicting the fact that $\Pi '$ is a shortest path. Hence the path $(w, v_t' + \mathbf e + \mathbf e', w')$ lies in $N_2(v_t') {\setminus } \Pi '$. The second possibility is that $w = v_t' - \mathbf e$ and $w' = v_t' + \mathbf e$ for some $\mathbf e \in \{\pm \mathbf e_j: j \le d\}$ which we can assume, without loss of generality, to be $\mathbf e_1$. Let $\mathbf e \in \{\pm \mathbf e_j: j = 2, 3\}$ and consider the path $(w, w + \mathbf e, v_t' + \mathbf e, w' + \mathbf e, w')$ in $N_2(v_t')$. If this path intersects $\Pi '$, then our previous argument yields that $v_t' + \mathbf e \in \Pi '$. Since $\Pi '$ is a self-avoiding path, it cannot contain more than 2 neighbors of $v_t'$ and thus the path $(w, w + \mathbf e, v_t' + \mathbf e, w' + \mathbf e, w')$ lies in $N_2(v_t') {\setminus } \Pi '$ for some $\mathbf e \in \{\pm \mathbf e_j: j = 1, 2\}$.

Thus it only remains to deal with the vertices adjacent to $v_1$ and $v_2$ which do not lie in $\Pi '$ or ${\mathcal {C}}_{\mathbf n_1 + \mathbf n_2}(S)$. Unfortunately it may not be always possible to connect a pair of such vertices by a path in $\{v_{d_1}'\}\cup N_2(\Pi ') {\setminus } \Pi '$ and in those cases we need to modify $\Pi '$. Below we discuss these cases based on all possible values of $d_\infty :=\Vert v - u\Vert _\infty$ and $d_1$. One observation that will be particularly useful is that $N_1(v_2')$ and ${\mathcal {C}}_{\mathbf n_1 + \mathbf n_2}(S)$ are disjoint. Thus we only need to connect any vertex in $N_1(v_1') {\setminus } (\Pi ' \cup {\mathcal {C}}_{\mathbf n_1 + \mathbf n_2}(S))$ to a vertex in $N_1(v_t') {\setminus } \Pi '$ for some $t \ge 2$ and similarly any vertex in $N_1(v_2') {\setminus } \Pi '$ to a vertex in $N_1(v_t') {\setminus } \Pi '$ for some $t \ge 3$ using a path in $N_2(\Pi ') {\setminus } (\Pi ' \cup {\mathcal {C}}_{\mathbf n_1 + \mathbf n_2}(S))$. As a final remark before we go to the details, let us mention that when $d_1 = 1$ or when $d_\infty = 2$ and $d_1 = 2$, it is not difficult to see that either $\Pi '$ or $(v_0', v_1')$ satisfies the items of the lemma. Hence, we only focus on the other cases.

*Case 1.*$$d_\infty \ge 3$$.

In this case we can choose $\Pi '$ so that $v_t' = v_0' + t \mathbf e$ for every $t \le 3$ and some $\mathbf e \in \{\pm \mathbf e_j: j \le d\}$. Without loss of generality, we assume that $\mathbf e = \mathbf e_1$. Notice that for any vertex *p* in $N_1(v_1') {\setminus } (\Pi ' \cup \mathcal C_{\mathbf n_1 + \mathbf n_2}(S))$, the vertex $p + \mathbf e_1$ lies in $N_1(v_1' + \mathbf e_1) {\setminus } \Pi ' = N_1(v_2'){\setminus } \Pi '$ and hence the edge $\{p, p + \mathbf e_1\}$ lies in $N_1(\Pi ') {\setminus } (\Pi ' \cup {\mathcal {C}}_{\mathbf n_1 + \mathbf n_2}(S))$ as well. Similarly any vertex in $N_1(v_2') {\setminus } \Pi '$ is either a neighbor of $v_4'$ (when $v_4' = v_3' \pm \mathbf e_j$ for some $j > 1$) or has a neighbor in $N_1(v_2' + \mathbf e_1) {\setminus } \Pi ' = N_1(v_3') {\setminus } \Pi '$ (when $v_4' = v_3' + \mathbf e_1$). These together imply that the items of Lemma 5.2 hold for $\Pi _{\mathbf B} = (v_0', v_1', \ldots , v_{t}')$, where $t = \min \{t' \le d_1: v_{t'}' \in {\mathcal {C}}_{\mathbf n_1}(y)\}$.

*Case 2.*$$d_\infty = 2$$ and $$d_1 \ge 3$$.

This is the most involved case. Here, we can assume without loss of generality that $v_t' = v_0' + t \mathbf e_1$ for $t \le 2$ and $v_3' = v_2' + \mathbf e_2$. Just like in Case 1, any vertex in $N_1(v_1') {\setminus } (\Pi ' \cup {\mathcal {C}}_{\mathbf n_1 + \mathbf n_2}(S))$ has a neighbor in $N_1(v_2') {\setminus } (\Pi ' \cup {\mathcal {C}}_{\mathbf n_1 + \mathbf n_2}(S))$. Also notice that for any vertex *p* in $N_1(v_2') {\setminus } (\Pi ' \cup {\mathcal {C}}_{\mathbf n_1 + \mathbf n_2}(S))$ other than $v_2' - \mathbf e_2$, the vertex $p + \mathbf e_2$ lies in $N_1(v_3') {\setminus } \Pi '$. Hence we only need a separate treatment for $v_2' - \mathbf e_2$, i.e. when it does not lie in ${\mathcal {C}}_{\mathbf n_1 + \mathbf n_2}(S)$. To this end we consider several sub-cases based on the neighboring vertices of $v_2' - \mathbf e_2$ and the value of $d_1$.

*Case 2-a.*$v_2' - \mathbf e_2 + \mathbf e \notin {\mathcal {C}}_{\mathbf n_1 + \mathbf n_2}(S)$*for some *$\mathbf e \in \{\pm \mathbf e_j: j \le d\} {\setminus } \{-\mathbf e_1, \pm \mathbf e_2\}$. Notice that $v_2' + \mathbf e \in N_1(v_2') {\setminus } \Pi '$. Since $N_1(v_2')$ does not intersect ${\mathcal {C}}_{\mathbf n_1 + \mathbf n_2}(S)$, it follows that the path $(v_2' - \mathbf e_2, v_2' - \mathbf e_2 + \mathbf e, v_2' + \mathbf e)$ lies in $N_2(\Pi ') {\setminus } (\Pi ' \cup {\mathcal {C}}_{\mathbf n_1 + \mathbf n_2}(S))$. Now $v_2' + \mathbf e$, being a neighbor of $v_2'$ other than $v_2' - \mathbf e_2$, has a neighbor in $N_1(v_3') {\setminus } \Pi '$ as we already saw above. Thus, like Case 1, we may choose $\Pi _{\mathbf B} = (v_0', v_1', \ldots , v_{t}')$ for $t = \min \{t' \le d_1: v_{t'}' \in {\mathcal {C}}_{\mathbf n_1}(y)\}$.

*Case 2-b. *$v_2' - \mathbf e_2 + \mathbf e \in {\mathcal {C}}_{\mathbf n_1 + \mathbf n_2}(S)$*for each*$\mathbf e \in \{\pm \mathbf e_j: j \le d\} {\setminus } \{-\mathbf e_1, \pm \mathbf e_2\}$*and*$d_1 > 3$. Let us modify $\Pi '$ slightly to obtain a new path $\Pi '' :=(v_2' - \mathbf e_2 + \mathbf e_1, v_2' + \mathbf e_1, v_2' + \mathbf e_2 + \mathbf e_1, v_3', \ldots , v_{d_1}')$ and ${\mathcal {C}}_{\mathbf n_1 + \mathbf n_2}(S)$ restricted to the box of side-length 6*dn* with the same center as $\mathbf B$ and *u*. In the same spirit as in the case of $\Pi '$, the only problematic vertex in $N_2(\Pi '') {\setminus } (\Pi '' \cup {\mathcal {C}}_{\mathbf n_1 + \mathbf n_2}(S))$ is $(v_2' + \mathbf e_2 + \mathbf e_1) + \mathbf e_1 = v_3' + 2\mathbf e_1$. Hence we can apply the argument from Case 2-a to $\Pi ''$*unless*$v_3' + 2\mathbf e_1 + \mathbf e \in {\mathcal {C}}_{\mathbf n_1 + \mathbf n_2}(S)$ for each $\mathbf e \in \{\pm \mathbf e_j: j \in [d]\} {\setminus } \{\pm \mathbf e_1, - \mathbf e_2\}$. But in that case, since $d_1 > 3$, there must be a vertex of ${\mathcal {C}}_{\mathbf n_1 + \mathbf n_2}(S)$ within a graph distance of 2 of $v_4$ and thus at a graph distance strictly smaller than $d_1$ of *u* which contradicts the definition of $d_1$.

*Case 2-c.*$v_2' - \mathbf e_2 + \mathbf e \in {\mathcal {C}}_{\mathbf n_1 + \mathbf n_2}(S)$*for each *$\mathbf e \in \{\pm \mathbf e_j: j \le d\} {\setminus } \{-\mathbf e_1, \pm \mathbf e_2\}$*and *$d_1 = 3$. In view of our discussion in the previous subcase, the only problematic scenario is the following. We have that $v_t' = v_0' + t \mathbf e_1$ for $t \le 2$, $u = v_3' = v_2' + \mathbf e_2$ and $u + 2\mathbf e_1 + \mathbf e \in {\mathcal {C}}_{\mathbf n_1 + \mathbf n_2}(S)$ for each $\mathbf e \in \{\pm \mathbf e_j: j \in [d]\} {\setminus } \{\pm \mathbf e_1, -\mathbf e_2\}$. Now recall that *u* has at least two neighbors in ${\mathcal {C}}_{\mathbf n_1}(y)$ which, we claim, gives us in this case two vertices *w* and $w'$ in ${\mathcal {C}}_{\mathbf n_1 + \mathbf n_2}(S)$ and ${\mathcal {C}}_{\mathbf n_1}(y)$ respectively such that $w' = w + 2 \mathbf e$ for some $\mathbf e \in \{\pm \mathbf e_j: j \le d\}$ and $w'$ is within a distance 1 of $\mathbf{B}$. Then it is straightforward to construct $\Pi _{\mathbf B}$ from *w* and $w'$ as we already remarked before starting our case studies. In order to verify our claim, we need to consider three distinct possibilities for the two neighbors of *u* that lie in ${\mathcal {C}}_{\mathbf n_1}(y)$.

(i) $v_2' = u - \mathbf e_2 \in {\mathcal {C}}_{\mathbf n_1}(y)$. In this case we can choose $w = v_0'$ and $w' = v_2'$.
(iii) $u + \mathbf e_1 \in {\mathcal {C}}_{\mathbf n_1}(y)$. Here our choices are $w = v_2' - \mathbf e_2 + \mathbf e_1$ and $w' = u + \mathbf e_1$.
(iii) $u + \mathbf e \in {\mathcal {C}}_{\mathbf n_1}(y)$*for some *$\mathbf e \in \{\pm \mathbf e_j: j \le d\} {\setminus } \{\pm \mathbf e_1, -\mathbf e_2\}$. In this case we choose $w = u + 2\mathbf e_1 + \mathbf e$ and $w' = u + \mathbf e$.

*Case 3.*$$d_\infty = 1$$ and $$d_1 \ge 3$$.

In this case, let us assume without loss of generality that $v_1' = v_0' + \mathbf e_1$, $v_2' = v_1' + \mathbf e_2$ and $v_3' = v_2' + \mathbf e_3$. Notice that any vertex *p* in $N_1(v_1') {\setminus } (\Pi ' \cup {\mathcal {C}}_{\mathbf n_1 + \mathbf n_2}(S))$ other than $v_1' - \mathbf e_2$ has a neighbor in $N_1(v_2') {\setminus } \Pi '$, namely $p + \mathbf e_2$. Also noting that the vertices $v_1' - \mathbf e_2 + \mathbf e_3$ and $v_1' + \mathbf e_3$ lie in $N_2(v_3){\setminus } \Pi '$ and $N_1(v_3){\setminus } \Pi '$ respectively, we deduce that $(v_1' - \mathbf e_2, v_1' - \mathbf e_2 + \mathbf e_3, v_1' + \mathbf e_3)$ is a path in $N_2(\Pi ') {\setminus } (\Pi ' \cup {\mathcal {C}}_{\mathbf n_1 + \mathbf n_2}(S))$ if $v_1' - \mathbf e_2 \notin {\mathcal {C}}_{\mathbf n_1 + \mathbf n_2}(S)$.

As to the vertices in $N_1(v_2') {\setminus } \Pi '$, we find by the same reasoning as in the analysis of Case 2-a that the only problematic scenario is $v_2' - \mathbf e_3 \notin {\mathcal {C}}_{\mathbf n_1 + \mathbf n_2}(S)$ and $v_2' - \mathbf e_3 + \mathbf e \in {\mathcal {C}}_{\mathbf n_1 + \mathbf n_2}(S)$ for every $\mathbf e \in \{\pm \mathbf e_j: j \le d\} {\setminus } \{-\mathbf e_2, \pm \mathbf e_3\}$. However, in this scenario we would have $\Vert (v_2' - \mathbf e_3 + \mathbf e_2) - u\Vert _1 = d_1$ whereas $\Vert (v_2' - \mathbf e_3 + \mathbf e_2) - u\Vert _\infty = 2$, thus reducing the problem to Case 2 with $v_0' = v_2' - \mathbf e_3 + \mathbf e_2$, which belongs to ${\mathcal {C}}_{\mathbf n_1 + \mathbf n_2}(S')$ and is within a distance of at most 3*dn* of the center of $\mathbf{B}$.

*Case 4.*$$d_\infty = 1$$ and $$d_1 = 2$$.

Let us assume without loss of generality that $u = v_0' + \mathbf e_1 + \mathbf e_2$ and $v_1' = v_0' + \mathbf e_1$. In this case we only need to connect any vertex in $N_1(v_1') {\setminus } (\Pi ' \cup {\mathcal {C}}_{\mathbf n_1 + \mathbf n_2}(S))$ to a vertex in $N_1(u) {\setminus } \Pi '$. To this end notice that any vertex *p* in $N_1(v_1') {\setminus } (\Pi ' \cup {\mathcal {C}}_{\mathbf n_1 + \mathbf n_2}(S))$ other than $v_1' - \mathbf e_2$ has a neighbor $p + \mathbf e_2$ in $N_1(u) {\setminus } \Pi '$. Now if $v_1' - \mathbf e_2 \notin {\mathcal {C}}_{\mathbf n_1 + \mathbf n_2}(S)$ and $u + \mathbf e$ is a neighbor of *u* in ${\mathcal {C}}_{\mathbf n_1}(y)$ which is not $u + \mathbf e_2$ (recall that there are at least two of them), then it is easy to see that either the path $(v_1' - \mathbf e_2, v_1' - \mathbf e_2 + \mathbf e, v_1' + \mathbf e)$ lies in $N_2(\Pi ') {\setminus } (\Pi ' \cup {\mathcal {C}}_{\mathbf n_1 + \mathbf n_2}(S))$ or there exists a vertex *w* in ${\mathcal {C}}_{\mathbf n_1 + \mathbf n_2}(S)$ satisfying $w = (u + \mathbf e) + 2\mathbf e'$ or $w = (u + \mathbf e) + \mathbf e'$ for some $\mathbf e' \in \{\pm \mathbf e_j: j \le d\}$. In both cases, the choice of $\Pi _B$ is clear. $\quad \square$
